# Chimpanzees overcome the tragedy of the commons with dominance

**DOI:** 10.1038/s41598-018-28416-8

**Published:** 2018-07-10

**Authors:** Rebecca Koomen, Esther Herrmann

**Affiliations:** Max Planck Institute for Evolutionary Anthropology, Leipzig, Germany

## Abstract

Competition over common-pool resources (CPR) is a ubiquitous challenge for social animals. Many species face similar dilemmas, yet our understanding of the evolutionary trajectory of CPR social strategies remains unexplored. Here, we provide a first look at the social strategies of our closest living relatives, chimpanzees (*Pan troglodytes*), in two novel resource dilemma experiments. Dyads of chimpanzees were presented with renewable resource systems, collapsible at a quantity-dependent threshold. Dyads had to continuously resist overconsumption to maximize collective gains. In study 1, dyads of chimpanzees sustained a renewing juice source. Inequality of juice acquisition between partners predicted sustaining success, indicating that one individual dominated the task while the partner inhibited. Dyads in study 2 fed together on accumulating carrot pieces but could end the accumulation any time by grabbing an immediate selfish source of carrots. Dyads with low tolerance were more successful at collectively sustaining the resource than highly tolerant dyads. Further, the dominant individual was more likely to cause collapse in dyads with low tolerance than dyads with high tolerance. These results indicate that chimpanzees use a dominance-based monopolisation strategy moderated by social tolerance to overcome the tragedy of the commons.

## Introduction

Humans are a profoundly cooperative species, yet the challenges posed by resource dilemmas may reveal the boundaries of our cooperative abilities. When resources are renewable, openly accessible, and subtractable - meaning any amount taken is subtracted from the total available for others at any given time –a common-pool resource (CPR) dilemma can result. Also referred to as the tragedy of the commons^1^, CPR dilemmas are socio-ecological interactions in which short-term selfish gains conflict with long-term group gains. To successfully sustain a CPR over the long-term, actors must limit their individual use, while enforcing the same on potential free-riders.

From mass extinctions^2^ to global climate change^3^, evidence of the challenges CPR dilemmas pose for humans abounds^1,4^. However, humans have demonstrated strategies to successfully avoid the tragedy of the commons^5–7^. Integrating two decades of empirical and theoretical CPR research, Agrawal^8^ identifies fairness of resource allocation to be one of the most important predictors of successful CPR strategies. In CPR dilemmas, fairness is often structured by equality. The importance of this simple resource distribution heuristic has been observed in field studies comparing the success of economically heterogeneous groups^9^ and replicated in experiments which manipulate resource access for some participants leading to a breakdown of success^10–12^. In a recent study with 6-year-old children, equality of resource acquisition between partners predicted success for pairs sustaining a CPR^13^.

A wide range of species, from fish^14^ and plants^15^ to parasites^16^ and microbes^14^ have been found to engage in competitive CPR scenarios. These species are thought to use non-social-cognitive mechanisms to resolve CPR dilemmas, such as kin selection, non-costly punishment^17^, and ecological feedback with diminishing returns^18^.

Despite the ubiquity and large-scale risks of CPR dilemmas, social strategies for sustaining CPRs have only been experimentally explored in human adults^19^ and recently in children^13^. As one of our closest living relatives, chimpanzees (*Pan troglodytes*) are a fitting comparative species for an investigation into the evolutionary origins of human CPR strategies. Like humans, chimpanzees live in complex social groups characterized by high levels of both cooperation and competition for resources^20^, and appear to also over-harvest resources in their natural environments, e.g.: chimpanzees in the Ngogo community hunt red colobus monkeys at unsustainable rates^21,22^, leading to the near local extinction of this species.

Chimpanzees have been shown to exhibit a set of social and cognitive skills likely to aid in CPR comprehension. For example, the necessity to limit one’s short-term resource consumption^19,23^ requires the ability to self-impose delay of gratification (DoG). Chimpanzees have shown this ability in tasks that involve waiting for a large reward in lieu of an immediately available, smaller reward^24–29^. In a paradigm in which food slowly accumulates, chimpanzees can maximise food collection by inhibiting reaching for the accessible food - which stops the accumulation mechanism - until all food has accumulated^27–29^. Moreover, chimpanzees can collectively exercise self-control in an interdependent token exchange task with a partner to accumulate individual food rewards^30^. Chimpanzees are also capable of mentally monitoring sequentially presented food in order to distinguish large from small quantities over time^31^. Further, chimpanzees have shown abilities for social coordination^32^, (even when payoffs are unequal^33^), collective action^34–36^, and DoG in a social context^29,30^.

Where children^13^ and adults^6,11^ succeed at overcoming CPR dilemmas with fairness, it is unlikely that chimpanzees’ social strategies in comparable dilemmas will show equitable patterns of resource distribution. Few studies have convincingly shown that fair distributions are important to chimpanzees^37–39^. For example, chimpanzee proposers in an ultimatum game preferentially choose the selfish option and, unlike humans, partners accept all non-zero offers^40^. These studies suggest that chimpanzees will not apply a social strategy involving fairness when faced with a CPR.

Here, we present two novel experimental paradigms (studies 1 & 2) for exploring the behavioural strategies of chimpanzees in a CPR dilemma. Both studies were designed with a temporally, socially, and causally simplified experimental paradigm to highlight the most fundamental aspects of a real-world CPR dilemma: outcome interdependence in a temporal resource dilemma in which actors have to continuously resist over-consumption in order to maximize collective gains^41^. The aim of both studies was to address the following questions: 1) Can chimpanzees individually sustain a CPR system even in a social context? 2) Can dyads of chimpanzees collectively sustain a CPR system? And, if yes, 3) what social patterns emerge in their strategies?

## Study 1: Eternal Fountain of Juice

Study 1 presented chimpanzees with a renewable mango juice CPR system, comparable in mechanism and methods to a recent CPR study with 6-year-old children^13^, exploring the developmental roots of human social strategies for overcoming these dilemmas. Juice continuously dripped from a visible source into an apparatus where it was available for drinking ad libitum. The resource system included a quantity dependent collapse mechanism that allowed participants to continuously track the level of available juice relative to a collapse threshold: drinking juice beyond the threshold caused a visible collapse, after which no further juice was available. Subjects had to inhibit collapsing the resource to access all the juice. Dyads experienced the resource system in two conditions: one with interdependence, the collective condition (1 shared resource), and one without interdependence, the parallel condition (2 independent resources). Because we know that social facilitation can increase food acquisition rate when two chimpanzees are in close proximity to one another while engaged in the same food collection task^42,43^, we designed the parallel condition as a control to isolate the independent CPR behaviours of chimpanzees in the same social and physical configuration as the collective condition. The role of the parallel condition was therefore to ascertain whether or not chimpanzees were capable of independently sustaining the juice system, which involved learning to inhibit rapid drinking, under social conditions likely to increase resource consumption rate due to social facilitation. We predicted the collective condition would be more difficult for chimpanzees to sustain than the parallel condition, and that equal distribution of juice between partners would not underlie successful CPR strategies in the collective condition.

## Study 1: Methods

### Study 1 (and 2): Ethics

Study 1 (and 2) were non-invasive and strictly adhered to the legal requirements of the country in which they were conducted. An internal ethics committee at the Max Planck Institute for Evolutionary Anthropology approved the studies as well as the Uganda Wildlife Authority and the Uganda National Council for Science and Technology. Animal husbandry and research complied with the “PASA Primate Veterinary Healthcare Manual” and the policies of Chimpanzee Sanctuary & Wildlife Conservation Trust (CSWCT). All subjects in studies 1 & 2 were semi-captive chimpanzees at Ngamba Island (CSWCT), Lake Victoria, Uganda. All subjects came to the sanctuary as unrelated orphans as a result of the illegal bushmeat trade, were raised by humans together with peers (and in most cases with surrogate chimpanzee mothers) and lived together in one social group. The vast majority of subjects had access to a large tract of primary forest (38.5 hectares) throughout the day. All chimpanzees came back from the forest every evening and spent the night in indoor enclosures with hammocks (average 35 m2). All subjects voluntarily participated in the study and were never deprived of food or water for any reason. In addition to the food available in the forest and the four species-appropriate meals they were provided, all subjects received mango juice (study 1) and carrots (study 2) in their night rooms, used as our testing rooms. Water was available ad libitum in the night rooms.

### Study 1: Subjects

Three chimpanzees (1 male) took part in the experiment. Six additional subjects (3 males) provided baseline drinking rate data (see supplementary material for subject details).

### Study 1: Materials

**Figure 1:**
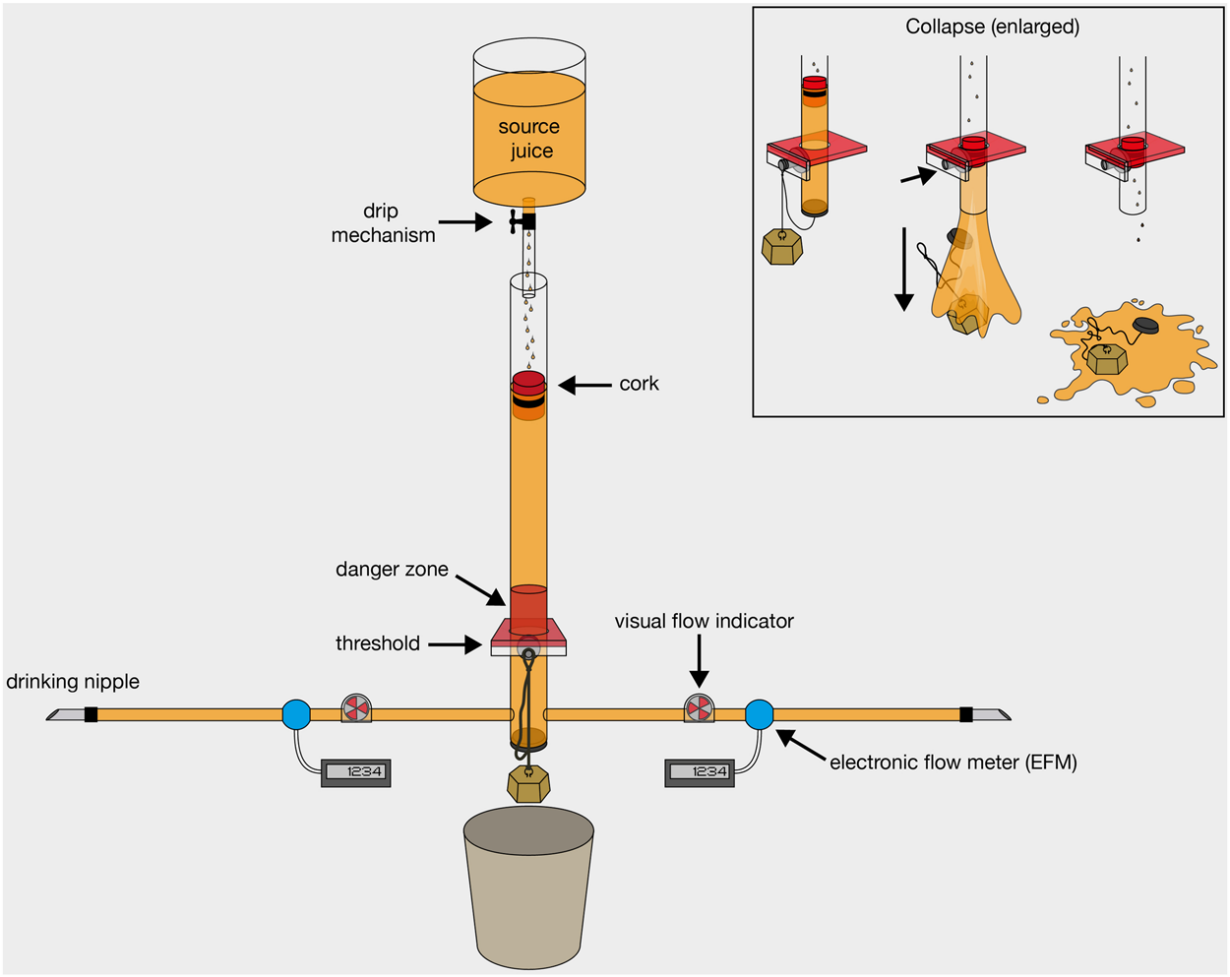
Study 1 apparatus mechanisms portrayed in detail.

The apparatus consisted of a transparent, vertically hanging cylinder (4 cm inner diameter, 77 cm length) with a plug and two hose attachments at the bottom (Fig. 1). At the end of each hose, a drinking nipple could be attached to the bars of two adjacent enclosures for chimpanzee access. Both subjects could therefore see one another while drinking and interact through the bars. Hanging above the cylinder was a transparent juice bottle (“source juice”, 1600 ml) with a tap mechanism that allowed the juice to drip into the cylinder. A red cork floated inside the cylinder to provide a visual cue of juice level and flow. When subjects drank, the cork sank downwards. If the cork reached a transparent, clearly marked red danger zone and threshold line an automatic magnet mechanism engaged, releasing the plug and causing all juice to fall into a bucket, inaccessible to subjects. Once this juice collapse occurred, any remaining source juice dripped directly into the bucket. Therefore, subjects had to inhibit from drinking the accessible 200 ml of juice below the threshold in order to avoid collapse. In the vast majority of sessions, the time for the full source juice to drip into the cylinder was 15–20 minutes. See supplementary material for a detailed description of the apparatus mechanisms.

Two flow wheels were inserted into each subject’s drinking hose: a visual flow indicator, and an electronic flow meter (hereafter “EFM”). The EFMs measured when and how much subjects drank in real time throughout experimental sessions. The visual flow indicators served as calibration gauges for EFM cross-validation, confirming onset and offset times of drinking bouts. See supplementary material for a detailed description of the EFM systems and calibration process.

### Study 1: Pre-test and familiarization

Six chimpanzees provided baseline drinking rate in pre-tests by drinking 1800 ml from the apparatus with the collapse potential removed.

A second group of 18 chimpanzees were individually familiarized with the apparatus. Three subjects learned to drink sustainably enough to access minimally 1 litre of source juice in the absence of the experimenter. Comprehension criterion was pre-defined as sustaining three subsequent sessions at this level of success. These three subjects moved on to the experimental phase; the other 15 were excluded. See supplementary material for a detailed description of the pre-tests and familiarization.

### Study 1: Design and procedure

**Figure 2:**
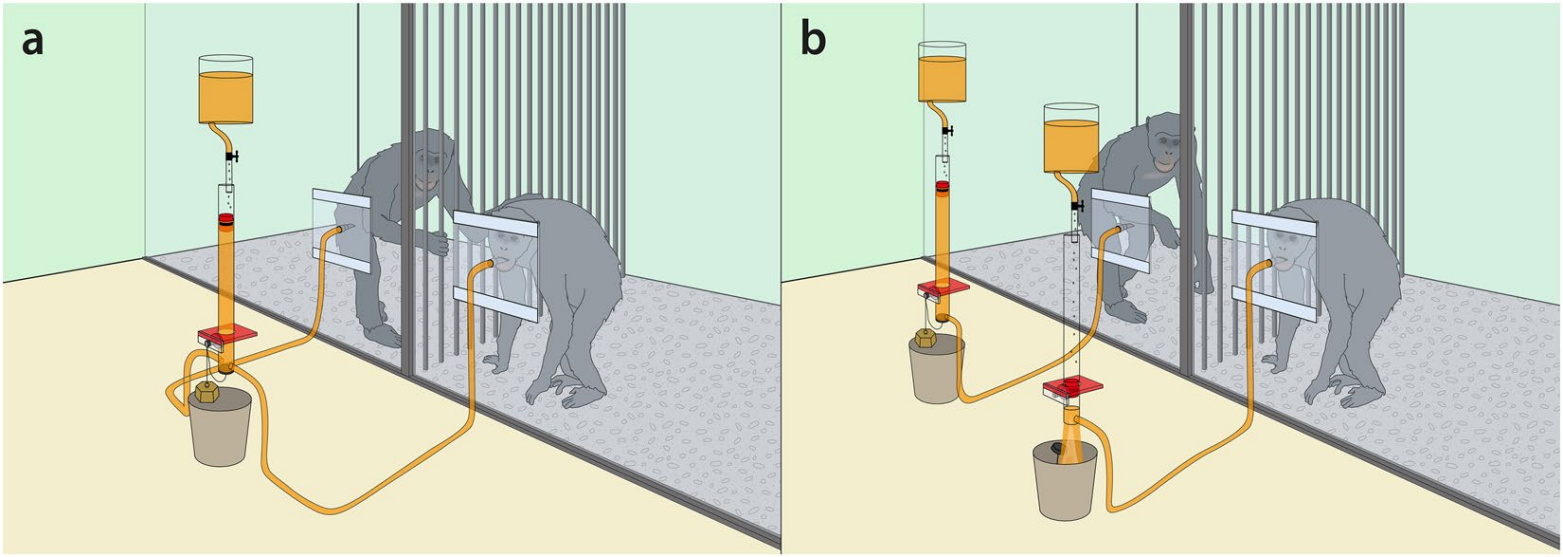
Study 1 setup in (**a**) the collective condition with one shared juice system, and (**b**) the parallel condition with two independent juice systems. The subject on the right has just collapsed her juice system.

The experiment consisted of a within-subjects design with two conditions: collective and parallel. In the collective condition, both subjects drank from a single shared juice system simultaneously (Fig. 2a). In the parallel condition (Fig. 2b), each subject had his or her own independently functioning juice system. In both conditions, partners sat next to one another in adjacent enclosures, close enough to reach an arm through the bars, but at no point were subjects able to control the juice access of their partners; both subjects always maintained juice access via their own drinking nipples irrespective of their partner’s behaviour. All juice systems functioned identically with the exception of the amount of juice in the cylinders at the start. To provide each subject with approximately 200 ml of juice at the start of sessions in both conditions, there was 400 ml of juice above threshold in the collective condition and 200 ml of juice above the threshold in each individual apparatus in the parallel condition. After the starting juice was consumed, the nature of the CPR dilemma task remained identical per apparatus across conditions.

Each dyad experienced a 12-day experimental cycle, with one session per day, in an A-B-B-A order: 3 sessions in the parallel condition, followed by 6 sessions in the collective condition, followed again by 3 sessions in the parallel condition. This experimental order was implemented to test for competitive carry-over effects from the collective condition to the parallel condition by testing for an order effect in the parallel condition.

In all sessions, the two subjects were first allowed into neighbouring test rooms. The experimenter began sessions by initiating dripping. Subjects were simultaneously given access to their drinking nipples by the experimenter and an assistant, both of whom immediately left the experimental area and did not return until the first collapse or to manually increase the drip rate (see supplementary material for details on manual drip rate increases and dyadic pairings).

### Study 1: Coding and analysis

Drinking rate pre-tests were coded for duration of drinking the full 1800 ml amount. For each experimental session, the following measures were coded: latency to 1^st^ collapse (hereafter ‘collapse latency’; measured in the parallel condition to the first of two possible collapse events), latency to drip stop to estimate full session drip rate (drip rate was variable between and within sessions: gravity caused drip rate to decrease gradually with time from session start to collapse), estimated drip rate within the first three minutes of sessions, all absence and presence bouts for each individual (defined as each subject being either >1 m away from, or <1 m proximity to the drinking nipples, respectively), as well as EFM indicated drinking bout onset times, offset times, and amounts per bout. We also coded for gestures: when an individual reached an arm or hand in the direction of their partner without culminating in a behaviour with a different function such as grabbing the bars, grooming, or holding something. Twenty percent of sessions were re-coded by another coder, blind to the predictions of the study and inter-rater reliability was high (see supplementary material for details).

To investigate differences in drinking behaviour between the parallel and the collective conditions, as well as how specific social behaviours contributed to sustaining success in the collective condition, we used a series of Generalized Linear Mixed Models (GLMM^44^), with significance set at (*p* < 0.05). See supplementary materials for all supporting data, model descriptions, and results.

## Study 1: Results and Discussion

The six subjects who took part in the drinking rate pre-test drank 1800 ml in an average of 7:31 (mm:ss; 3.99 ml/sec; 18 sessions, range 4:11–12:30, 95% CI ± 1:07) – less than half the amount of time it took for the full source juice to drip into the cylinder in experimental sessions. Thus, subjects had to drink at approximately 1/2 the preferred drinking rate to sustain the juice systems in the parallel condition. In the collective condition, both individuals would have to drink at approximately 1/4 of the preferred drinking rate in order to equitably sustain the system.

**Figure 3:**
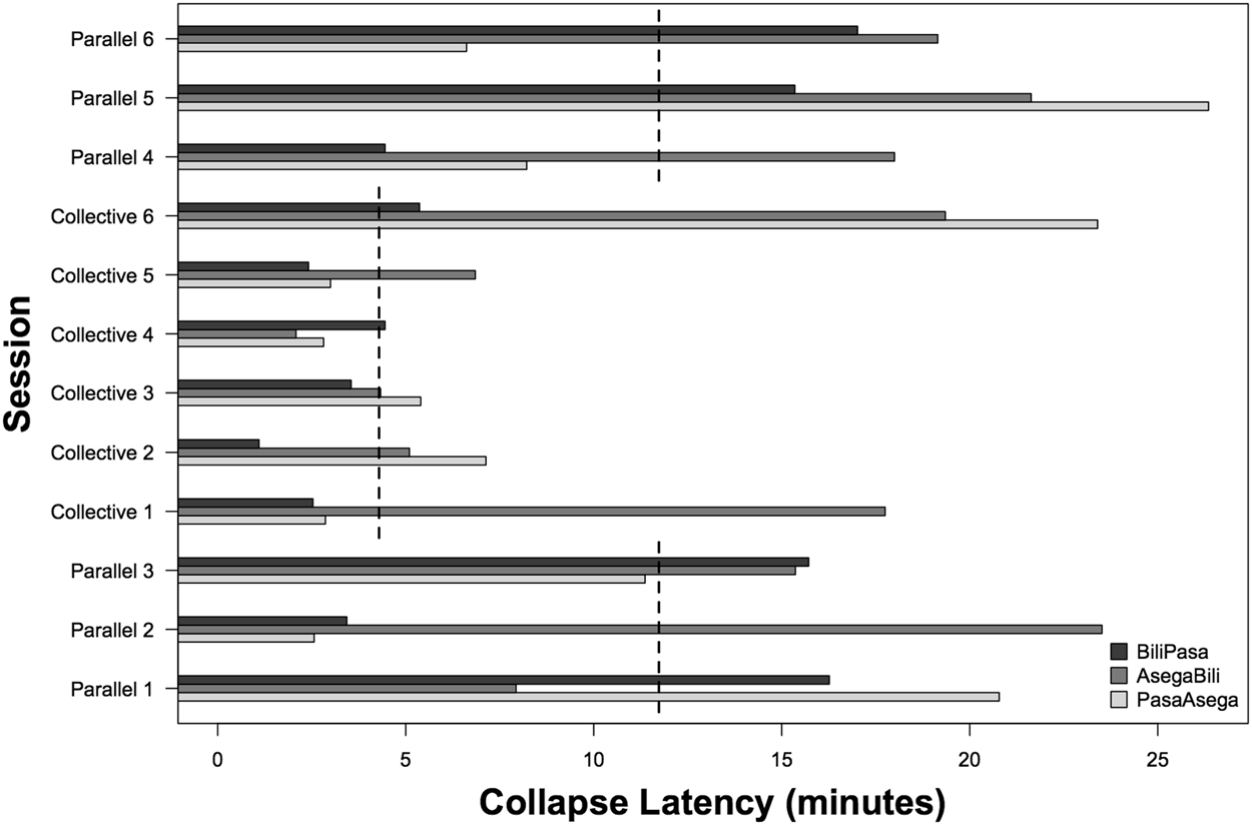
Collapse latencies (x-axis) of all sessions, plotted in chronological order of sessions received by dyad from bottom to top on the y-axis. Vertical dashed lines represent collapse latency predictions of the model for each condition. The collective condition was more difficult than the parallel condition, yet three of these collective sessions clearly exceeded predicted latencies for the CPR condition.

All three tested pairs of chimpanzees successfully sustained the juice system in the parallel condition: in all 18 parallel sessions, at least one subject successfully sustained 100% of the drip juice, and in 10 of these sessions both individuals accessed 100% of the drip juice. Two of the three dyads were also able to collectively sustain the resource long enough to access 100% of the juice in three out of 18 collective condition sessions. The average collapse latency in the parallel condition was 14:06 (18 sessions, 95% CI ± 3:19), with a range from 2:34 to 26:21; the average collapse latency in the collective condition was 6:38 (18 sessions, 95% CI ± 3:00), with a range from 2:32 to 23:24 (see Fig. 3 for all collapse latencies). See supplementary material for experimental drip rate information.

We first tested the effect of condition, session number and drip rate on sustaining success, measured by latency to 1^st^ collapse, controlling for the random effects of dyad. The sample size for this test was 35 (one session dropped from the 36-sessions dataset due to a lack of drip rate data). We included an interaction between session number and condition with the prediction that increasing experience may differentially affect success in the two conditions (specifically, that the competition of the collective condition may moderate any observable learning effect across sessions relative to the parallel condition). According to this model, condition (χ2 = 6.93, df = 1, p < 0.01) significantly predicted success: dyads sustained the resource longer in the parallel condition than in the collective condition (Fig. 3). Neither session number (χ2 = 1.2, df = 1, p = 0.27) nor drip rate in the first three minutes (χ2 = 0.01, df = 1, p = 0.9) had an effect on sustaining success, which indicates that no pattern of learning was observable across sessions, and that successful behaviour was more flexible than had it been contingent on the rate of juice availability being stable across sessions.

Because we predicted subjects would not likely engage in equal distribution, we tested for an effect of juice acquisition inequality between partners on success in the collective condition only (17 sessions; one session dropped due to EFM technical failure). We found a trend (χ2 = 3.34, df = 1, p = 0.067) indicating that dyads sustained the CPR system longer when the amounts of juice drunk by partners differed furthest from an equal 50:50 split (see supplementary material for a visualisation of the data).

**Figure 4:**
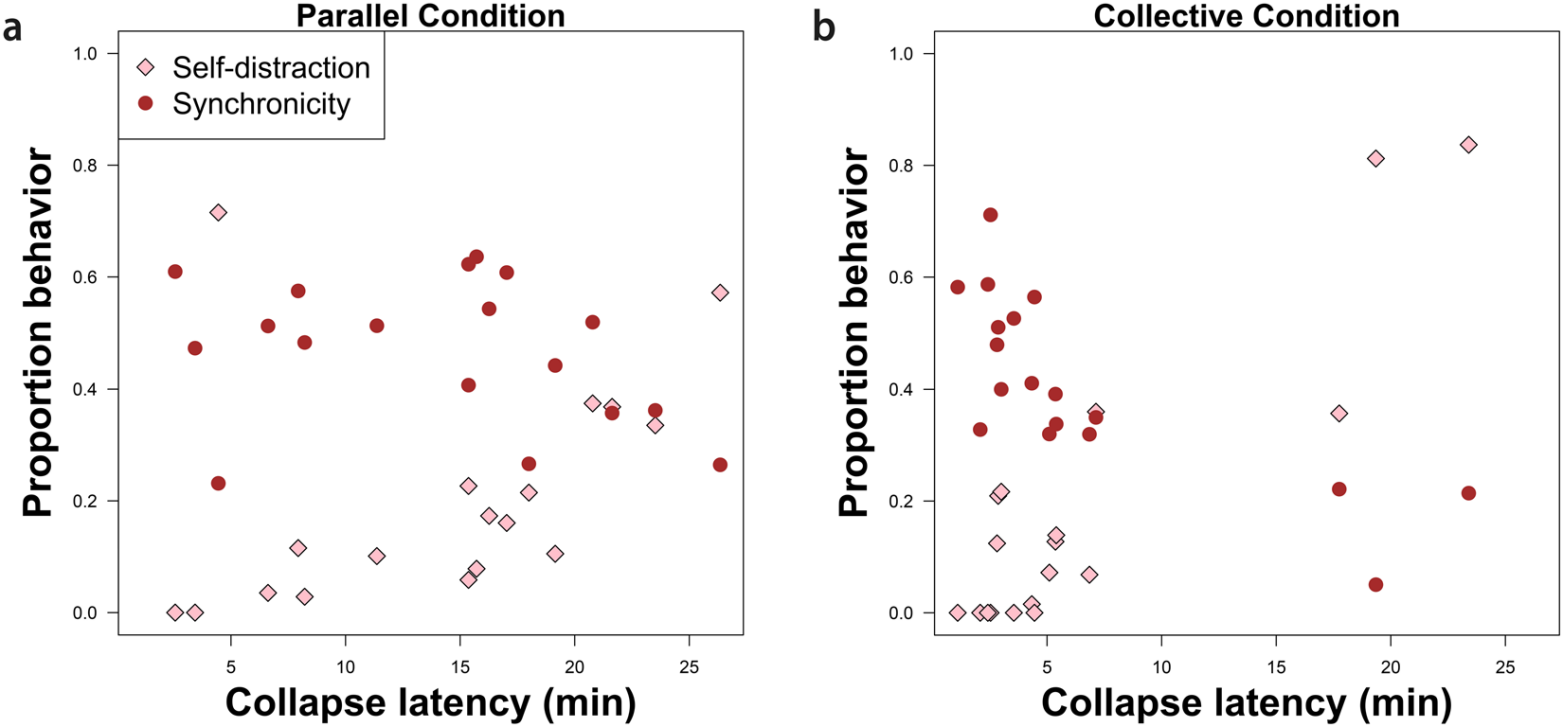
Per-session proportions of self-distraction (pink diamonds) and drinking synchronicity (red dots) as a function of collapse latency on the x-axis in (**a**) the parallel condition and, (**b**) the collective condition.

We investigated the role of self-distraction behaviour, defined as the combined duration of time either subject was absent from the drinking nipple area over the session duration (i.e. collapse latency; see supplementary material for analysis on effect of partner presence on individual drinking rate), as well as drinking synchronicity, defined as the proportion of time subjects in a dyad spent drinking simultaneously over the total time any subject spent drinking per session. Although it was not possible to test formally for the effect of these proportional behaviours on success (see supplementary material for details), self-distraction was more common in highly successful collective condition sessions than successful parallel condition sessions. The opposite was true for drinking synchronicity: subjects appeared less synchronous in highly successful collective condition sessions when compared to successful parallel condition sessions (Fig. 4).

**Figure 5:**
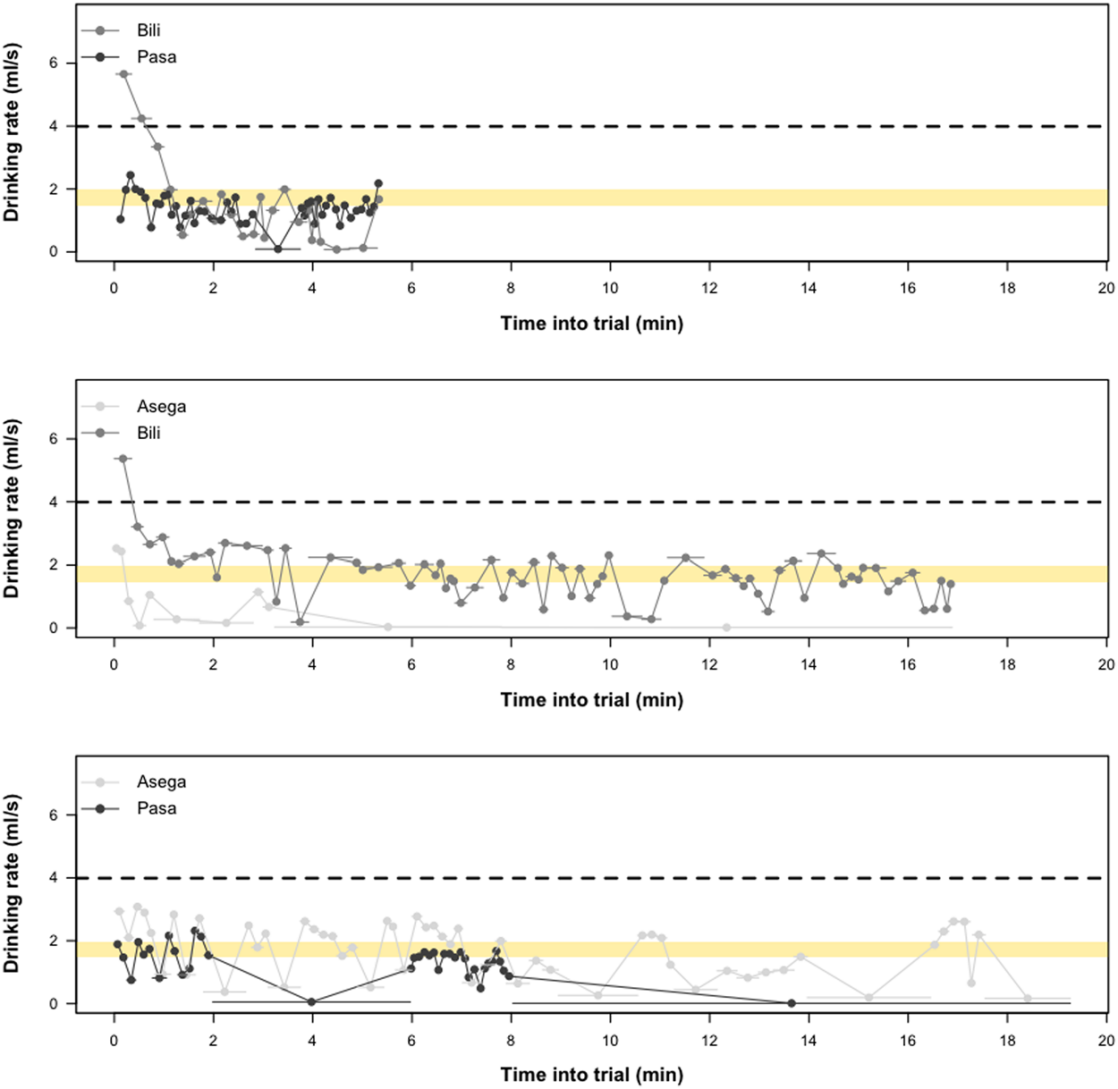
The individual drinking rates of each dyad’s most successful collective condition session (also last session of this condition for all dyads). The dashed line indicates the average drinking rate of pre-test subjects. The yellow bar indicates the range of the estimated juice drip rate from the source into the cylinder where it became available for drinking.

We also investigated variation in drinking rate within collective condition sessions with respect to the average drip rate as well as the average drinking rate of the pre-test subjects for whom no drinking inhibition was required. A visual inspection of the data from each dyad’s final session (Fig. 5) revealed a pattern in which one individual decreased his/her drinking rates to approach 0 ml/sec while the partner maintained a drinking rate hovering around the estimated drip rate (yellow bar), maximising his/her juice intake according to juice availability above the threshold at the expense of the partner.

A total of 11 gestures were observed, 10 of which occurred in the collective condition. All gestures were accompanied by a simultaneous gaze in the direction of the partner and all but four were also accompanied by a low volume vocalization. See supplementary material for a video of gesture occurrences, and an ethogram.

The overall pattern of results in study 1 is suggestive of an unequal, asynchronous dyadic strategy in which one individual periodically slows their drinking or abstains from taking part while the partner continues to maximize drinking rate in accordance with the drip rate. Dyadic partners in the most successful sessions drank the most unequal amounts of the shared resource, tended to drink at different times (asynchronously), and tended to leave the drinking area more. It remains an open question how inter-individual relationship characteristics affect this pattern.

Owing to the difficulty of the task, study 1 was limited to a very small sample size (N = 3). For this reason, study 2 was designed with simpler task comprehension demands, allowing for more participants with which we could investigate the effects of learning across sessions, as well as of social relationships – specifically the role of dominance within dyads and tolerance between dyads – on CPR dilemma success.

## Study 2: Common-Pool Carrots

In Study 2, dyads of chimpanzees were presented with a renewing, collapsible carrot system. For the renewal mechanism, we incorporated methods from previous DoG experiments^27^. Subjects could eat carrot pieces accumulating in a shared feeding area. At any time, subjects could collapse the carrot system by grabbing a wooden rod, baited with a small, immediate, selfish portion of carrot pieces. Collapsing the carrot system resulted in the continually accumulating carrot pieces falling outside of either subjects’ reach. As in study 1, dyads in study 2 experienced the carrot CPR system in two conditions: parallel and collective (see supplementary material for a detailed comparison of study designs). The prediction for study 2 was that successful CPR strategies would be mediated by dyadic social dynamics such as dominance and co-feeding tolerance (hereafter: tolerance).

## Study 2: Methods

### Study 2: Subjects

The pool of subjects for study 2 came from the same group as study 1. All three subjects from study 1 also took part in study 2 (the female-female dyad, Bili-Pasa, participated in both studies as a dyad). Thirty-one chimpanzees (15 males) participated in study 2. Eight subjects were dropped from initial familiarization; of the remaining 23, 17 were familiarized to criterion.

### Study 2: Materials

**Figure 6:**
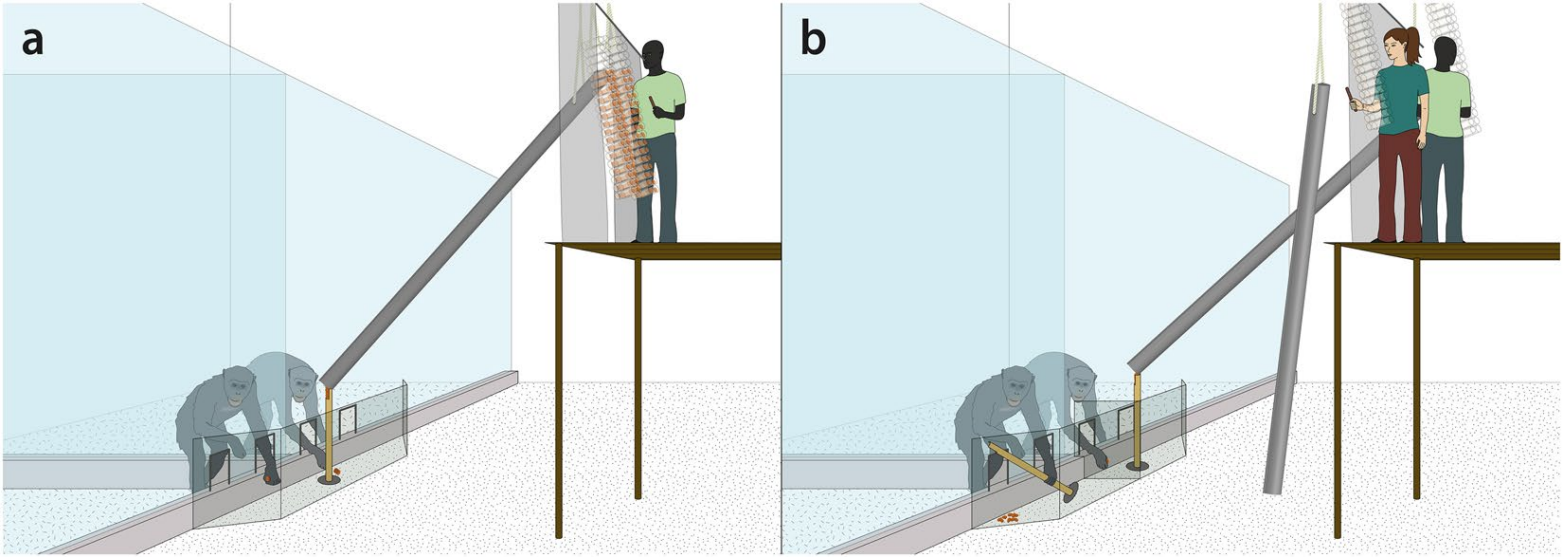
Study 2 setup in (**a**) the collective condition (E1 present but not pictured) and (**b**) the parallel condition in which the subject on the left has just collapsed her carrot system.

The carrot drop apparatus was set up outside the same two adjacent rooms as in study 1. The collective condition carrot system comprised a chute hanging from the ceiling (Fig. 6a). The lower end of the chute rested on top of a vertical wooden rod (3 cm diameter, 84 cm height;) in reach of both subjects outside their adjacent enclosures. Two experimenters stood on a raised platform (184 cm height) opposite the subjects’ rooms, behind a curtain. Hanging at the height of the platform were two carrot display sets comprised of 40 transparent tubes, each tube baited with two carrot pieces, hanging at an angle of 30° from vertical such that the subjects below could see the contents of the full array of display tubes at all times. Every 10 seconds, both experimenters dispensed two pieces of carrot into the chute (4 pieces total, using tools on sticks) where they would fall down audibly and land in a collective feeding area in front of the two adjacent rooms, equally accessible to both subjects through two feeding holes in front of each enclosure. Between dropping iterations, the experimenters used their tools to provide auditory cues to signal the impending arrival of the next carrot dropping iteration.

At the beginning of all experimental trials, the top of the rod holding up the chute was baited with eight carrot pieces. The only way to access these eight pieces was for one of the subjects to reach through one of their two feeding holes, grab the rod, and pull it toward the bars. Doing so, however, broke the connection between the chute and the rod, allowing the chute to drop to a vertical angle. This event represented resource collapse: all further dropping carrots fell outside the feeding area, inaccessible to subjects. In all familiarization and experimental test trials, carrot dropping iterations continued until the dispensing tubes on the platform were empty (40 iterations, 6:30 duration), regardless of the timing of collapse.

The apparatus setup in the parallel condition resembled the collective condition except the single system was split into two independently functioning systems (Fig. 6b). A second chute and rod were added with the curtain between them to create visual separation of each subject’s system, however both were still visible to both subjects at all times. A transparent divider was inserted into the collective feeding area to divide the carrot accumulation areas of each subject.

### Study 2: Pre-tests and familiarization

All 31 subjects received two initial familiarization sessions (phase 1) to establish baseline participation rates and skills in pulling the baited rod and collecting accumulating carrot pieces from the chute, after which eight subjects were dropped due to a lack of engagement with the apparatus or carrots.

The remaining 23 subjects received a series of familiarization sessions (phase 2) ad libitum until they reached criterion, defined as individually sustaining a full cycle of carrot renewal (40 dropping iterations of 2 pieces each) before pulling the baited rod (8 pieces) on two subsequent sessions. Seventeen subjects passed criterion; the remaining six were excluded, along with one of the 17 successful subjects for an even number of test subjects (N = 16). All three subjects from study 1 were included in the final subject pool. Dyads were then given pre-tests to determine dominance and tolerance (see supplementary material for pre-test and familiarization details).

### Study 2: Design and procedure

Study 2 followed a within-subjects design. The two conditions (collective and parallel) were counterbalanced for two groups, such that one group was tested in and A-B order, and the other a B-A order. Both groups had approximately equal aggregate tolerance scores to control for any interaction between order of conditions and tolerance. Dyads experienced eight trials in each condition: two trials per session, one session per day, across four sessions. Inter-trial intervals were approximately 10 minutes. All trials began with two free carrot pieces placed in front of each subject’s feeding holes. The first dropping iteration occurred when both subjects were allowed simultaneous entrance to the testing rooms, marking trial start. Trials ended after all 40 dropping iterations had been completed (6:30), irrespective of collapse latency.

### Study 2: Coding and analysis

Tolerance pre-tests were coded for inequality of food access, from which dyadic dominance was established, and for the latency of the subordinate to place an arm through the feeding hole nearest to his/her partner’s enclosure.

Experimental trials were coded for two dependent measures: collapse latency, defined as the duration of the trial from start to the time when the chute physically collapsed, and collapser identity (subordinate or dominant). Twenty percent of trials were re-coded by a second coder, blind to the predictions of the study. Inter-rater reliability was high (see supplementary material for details).

The sample size for the analyses was 128 collapse events (trials). Study 2 was analysed with the same statistical methods as study 1 (see supplementary materials for all supporting data, full model descriptions, and results). We first tested for the effects of condition, tolerance level, and trial number (as well as their interaction) on collapse latency. Additionally, we tested for the effects of condition and tolerance (and their interaction) on collapser ID (dominant or subordinate).

## Study 2: Results and Discussion

**Figure 7:**
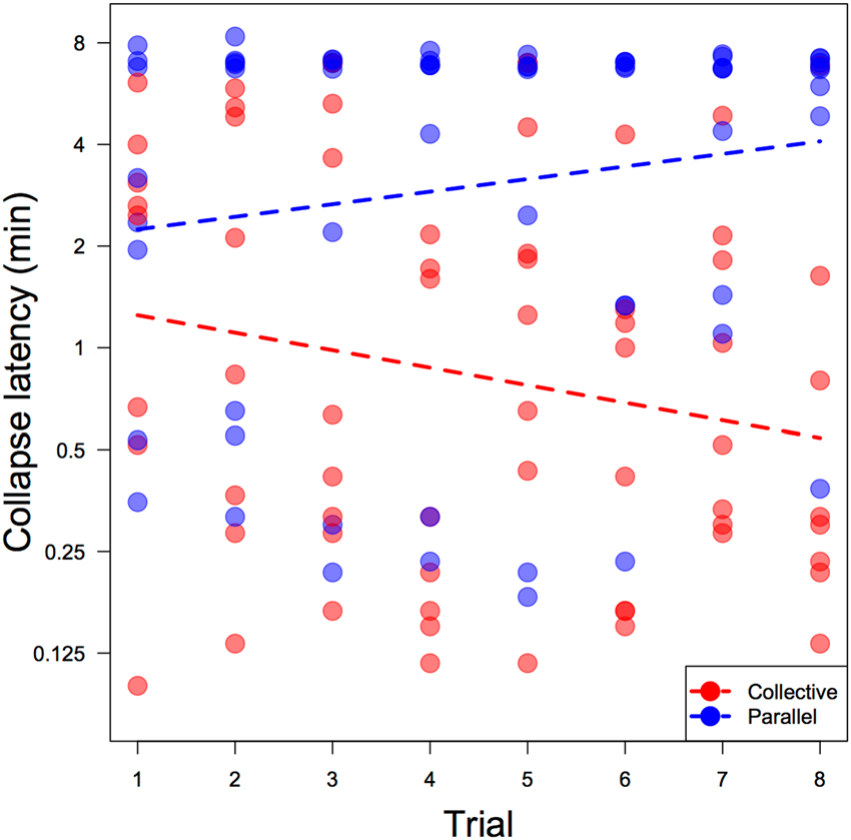
Effect of trial number and condition on collapse latency in minutes (displayed on a log-scale). Dyads improved collapse latency with experience in the parallel condition (blue) and shortened collapse latency with experience in the collective condition (red).

The average collapse latency in the parallel condition was 4:49 (64 trials, range 0:07–10:17, 95% CI ± 0:43), and 1:44 (64 trials, range 0:06–6:59, 95% CI ± 0:30) in the collective condition. Examining the effect of trial number, condition, and tolerance on collapse latency revealed two significant two-way interactions: between trial number and condition (χ2 = 6.99, df = 1, p < 0.01), and tolerance and condition (χ2 = 5.72, df = 1, p = 0.02). The first interaction indicated that dyads in the parallel condition increased their collapse latency with experience across trials, however this effect was different for dyads in the collective condition, in which collapse latency decreased with experience (Fig. 7).

**Figure 8:**
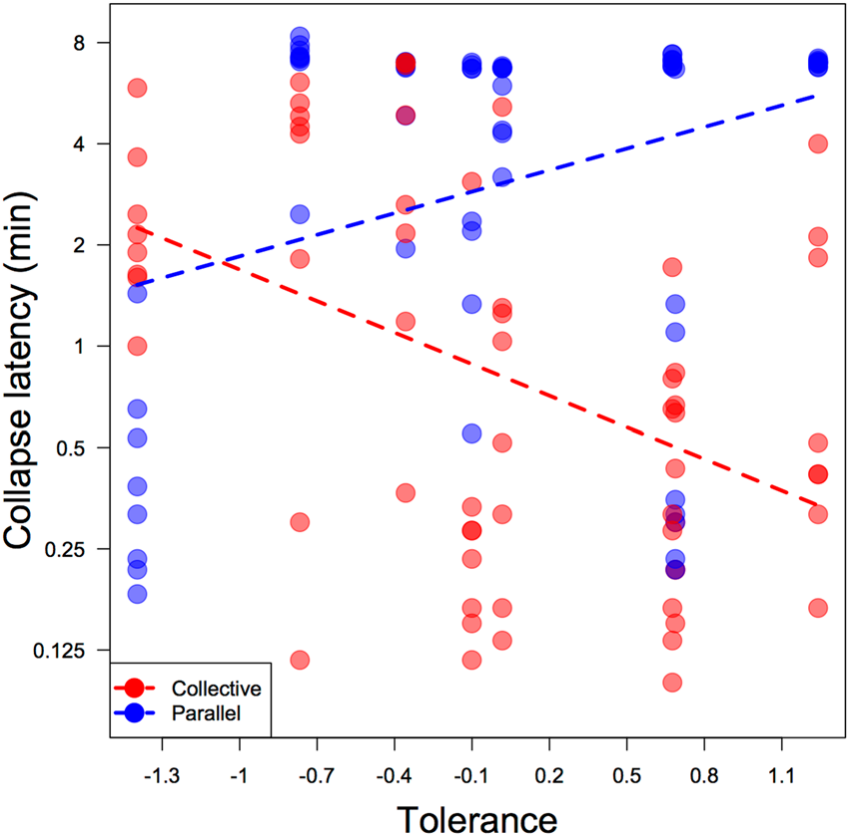
Effect of dyadic co-feeding tolerance and condition on collapse latency in minutes (displayed on log-scale). More tolerant dyads performed better in the parallel condition and worse than low tolerance dyads in the collective condition.

The second interaction revealed that tolerance had a differential significant effect on collapse latency according to condition: low tolerance dyads sustained the carrot system longer than high tolerance dyads in the collective condition. This effect was opposite in the non-competitive parallel condition (Fig. 8).

We also tested for effects of the interaction between tolerance and trial number on collapse latency. This interaction was non-significant (χ2 = 0.15, df = 1, p = 0.7), indicating that the ratcheting up of competition in the collective condition was not driven by high tolerance dyads any more than by low tolerance dyads.

**Figure 9:**
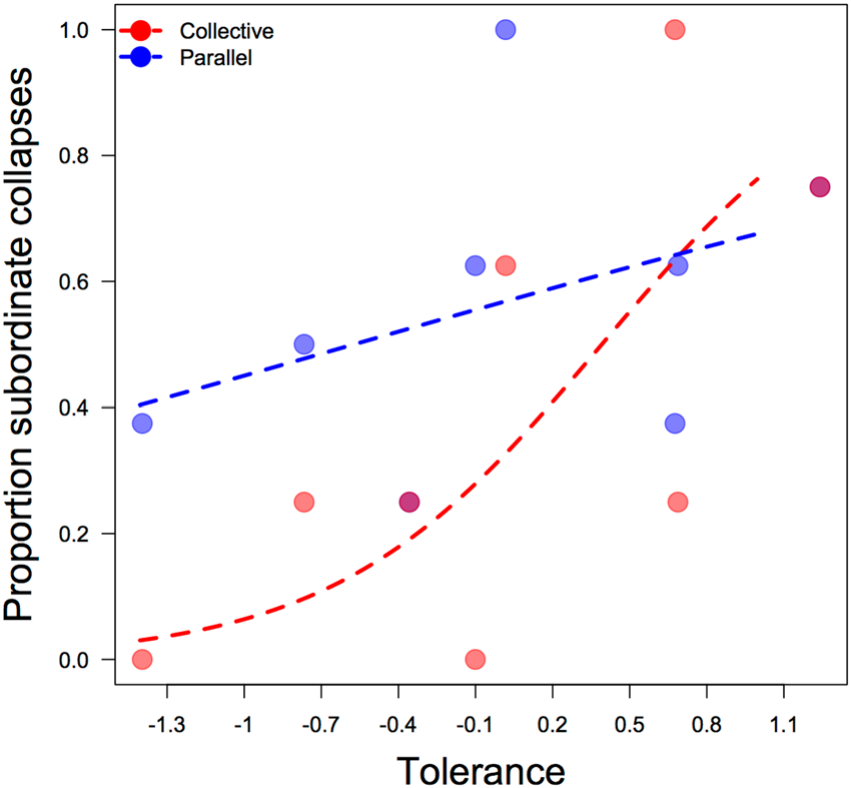
Effect of co-feeding tolerance and condition on the proportion of subordinate-caused collapses. The interaction between tolerance and condition showed a non-significant trend: far fewer subordinate individuals in low tolerance dyads caused collapse in the collective condition than in the parallel condition.

Looking at the effect of condition and tolerance level on the likelihood that the subordinate individual caused the collapse revealed an effect approaching significance for the full-null model comparison (χ2 = 7.54, df = 3, p = 0.057). This indicated that condition and tolerance level likely had an effect on the subordinate individuals’ propensity to cause collapse. The interaction between condition and tolerance level was indeed approaching significance (χ2 = 3.1, df = 1, p = 0.078). Although not significant at the 0.05 level, this result suggests that for low tolerance dyads, fewer subordinate individuals collapsed the CPR in the collective condition than the parallel condition (Fig. 9). In the collective condition, therefore, the dominant individuals appear to have taken control of the collapse mechanism more often in low tolerance dyads than in high tolerance dyads. As tolerance increased, so did the likelihood of a subordinate-caused collapse in both conditions.

Chimpanzee dyads in study 2 sustained the parallel systems longer than the collective system, and this difference became more extreme across trials as dyads gained experience in each condition. The fact that sustaining success decreased across collective condition trials indicates a ratcheting-up of competition in place of the learning effect in the parallel condition. This ratcheting-up of competition was shown in dyads across the tolerance spectrum and was therefore not driven by high tolerance dyads, who were less successful at sustaining the collective condition system than low tolerance dyads.

The overall pattern of results in study 2 is suggestive of an unequal, asymmetrical dyadic strategy in which the dominant individual of low tolerance dyads controls the collapse-causing resources while the subordinate partner inhibits from taking these resources. Dyads with high tolerance were presumably less successful in the collective condition because both partners were likely to cause collapse and were thus in competition with one another to be the first to grab the rod. Lower tolerance dyads sustained the collective resource longer than high tolerance dyads because, with lower tolerance, the dominant individual maintained more control over the collapse mechanism, to a certain extent mitigating the competition of the dilemma.

### Data Availability

Supporting data sets are available as supplemental files.

## General Discussion

By presenting dyads with two renewing, collapsible common-pool resource systems, we have shown that chimpanzees, like human adults^41^ and children^13^, are prone to the tragedy of the commons. In both studies 1 and 2, however, some pairs were able to avoid resource collapse. In study 1, two out of three dyads achieved 100% success in 25% of their collective condition trials; in study 2, half of the dyads reached 75% sustaining success or higher in 28% of their collective trials. In both studies, success was higher when dyads worked in parallel than when they accessed the CPR collectively.

Study 1 showed that dyads were better able to collectively sustain the CPR when resource acquisition between partners was unequal, in direct contrast to the successful strategies of 6-year-old children^13^, who were most successful when they equitably distributed the resource, and in contrast to successful adult human strategies, often underpinned by norms of fairness and equality^6^. The inequality in successful trials appears to be caused by asynchronous drinking and self-distraction behaviours. Chimpanzees have been shown to cope with impulsivity by self-distracting in independent tasks requiring DoG^28^. Chimpanzees in study 1 showed a tendency to self-distract by walking away from the drinking area more often during highly successful trials in the collective condition than in the parallel condition or in unsuccessful collective condition trials. Self-distraction may contribute to success, in that it decreases both socially facilitated drinking responses and the number of individuals actively engaged in drinking at any time (see supplementary material for further analyses and discussion on the effect of condition and partner presence on drinking rate).

In study 2 a learning effect allowed dyads to increase their success in the parallel condition over time but the opposite was true for the collective condition; this result suggests a ratcheting-up of competition in the CPR dilemma. In a CPR paradigm comparable to study 1, children became increasingly successful across three collective condition trials^13^, a reverse finding to that observed in chimpanzees in study 2. Moreover, higher tolerance led to higher success in the parallel condition but again the opposite was true for the collective condition. This result replicated the findings of study 1 which showed that inequality of juice access predicted CPR success. In study 2, inequality of access to shared food in tolerance pre-tests was one of two measures embedded in each dyad’s tolerance level. Dyads who shared unequally in the pre-test were also more likely to succeed in the CPR dilemma together. Because inequality underpinned success in study 1, and study 2 showed that low tolerance (based on inequality of food access) predicted success, these two studies together suggest that inequality of resource access is important for successful chimpanzee strategies in overcoming a common-pool resource dilemma. These findings are striking in comparison to the equality that predicted successfully sustained CPR dilemmas in children in a comparable experiment^13^.

The beneficial effect of face-to-face communication in human CPR dilemmas has been observed in field studies and experimentally replicated^10^. While not required for success, the gestural events observed in study 1’s collective condition could, however, be an indication of communicative intent, particularly because all gestures were also accompanied by a simultaneous gaze in the direction of the partner and, in most cases, a low volume vocalization. All collective condition gestures also occurred while the cork was in the danger zone. Though evidence of chimpanzee cooperative communication is scant – their gestures being mostly individualistic and imperatively motivated^45,46^ – the gestures in study 1 may indicate that chimpanzees can, in some cases, spontaneously elicit communicative strategies in novel cooperative contexts.

In line with previous findings^34,47^, tolerance in study 2 moderated the strength of the binary difference between the dominant and subordinate partner. Because both partners in high tolerance dyads showed a strong propensity to participate, success at sustaining the CPR in study 2 was lower than it was for less tolerant dyads.

Taken together, the results of study 1 and 2 suggest that successful chimpanzee CPR strategies involve unequal access to the resource, regulated by social dominance, mediated by tolerance between partners. Dominance asymmetries allow many animal species to resolve conflicts over resources by signalling to subordinate individuals that backing down could help avoid aggressive escalation with dominant individuals^48^. For chimpanzees, when tolerance is low enough that the subordinate’s full participation entails risk, the dominant chimpanzee in a dyadic CPR is able to monopolize the collapse-causing resources, thereby mitigating the competition of the dilemma. When tolerance is high, both the subordinate and the dominant individual participate in the dilemma: competition is higher, and success is lower. Whereas fairness norms regarding equal resource allocation facilitate successful human CPR strategies, equality of resource access within chimpanzee pairs led to faster resource collapse.

The monopolization observed in chimpanzee strategies closely approximates the outcome of what economists have historically called for in human CPR management policies: privatisation of natural resources via “mutual coercion,” because “injustice is preferable to total ruin”^1^ (p.1247). In order to avoid the tragedy of the ‘unmanaged’ commons, theorists have traditionally argued that sub-sections of the population must be excluded from free access^1,49^. The psychological mechanisms upholding the dominance-based inequality strategies of chimpanzees in studies 1 & 2, and the environmental policies proposed by early economists likely differ, particularly to the extent that chimpanzees always maintained physical access to the resource but inhibited along hierarchical lines, as opposed to being forcibly prevented from harvesting a resource, as is often the case with human exclusion. However, the outcome is comparable nonetheless: partial or full exclusion of some resource users for the sake of maximizing resource consumption over time for other users. By excluding participants, the resource itself is no longer fully open-access and is therefore no longer subject to the same competitive strain as a CPR dilemma. This is a legitimate means of overcoming the dilemma.

The paradigms presented here provide the first evidence that chimpanzees can, in some cases, collectively overcome the tragedy of the commons. This is also the first evidence that chimpanzees achieve success with demonstrably different social strategies than human adults and children, suggesting that our reliance on fairness in CPR dilemmas may be the result of newly derived social-cognitive tools, unique to our species.

Future studies should expand these paradigms beyond the dyad to group-wide behaviour over longer time intervals, as well as cross-group comparisons, allowing for a more comprehensive analysis of how social dynamics and social climate structure behaviour in CPR dilemmas. Finally, to strengthen our understanding of human CPR strategies we must expand these experimental paradigms to other species and environments.

## Electronic supplementary material

Eternal Fountain of Juice Gestures

Main supplementary materials

Dataset 1

Dataset 2

Dataset 3

Dataset 4
