# Supplementary material for: Chimpanzees overcome the tragedy of the commons with dominance
Source: Sci Rep. 2018 Jul 10;8:10389. doi: 10.1038/s41598-018-28416-8 (PMC6039489; doi:10.1038/s41598-018-28416-8)
Supplement: Supplementary file 2 — Main supplementary materials [file 41598_2018_28416_MOESM2_ESM.docx]

Chimpanzees overcome the tragedy of the commons with dominance

Rebecca Koomen & Esther Herrmann

Max Planck Institute for Evolutionary Anthropology

**Supplementary Material**

**Study 1 (& 2): Ethics**

Study 1 & 2 were non-invasive and strictly adhered to the legal requirements of the country in which they were conducted. An internal ethics committee at the Max Planck Institute for Evolutionary Anthropology approved the studies as well as the Uganda Wildlife Authority and the Uganda National Council for Science and Technology. Animal husbandry and research complied with the “PASA Primate Veterinary Healthcare Manual” and the policies of Chimpanzee Sanctuary & Wildlife Conservation Trust (CSWCT). All subjects in studies 1 & 2 were semi-captive chimpanzees at Ngamba Island (CSWCT), Lake Victoria, Uganda. All subjects came to the sanctuary as unrelated orphans as a result of the illegal bushmeat trade, were raised by humans together with peers (and in most cases with surrogate chimpanzee mothers) and lived together in one social group. The vast majority of subjects had access to a large tract of primary forest (38.5 hectares) throughout the day. All chimpanzees came back from the forest every evening and spent the night in indoor enclosures with hammocks (average 35 m^2^). All subjects voluntarily participated in the study and were never deprived of food or water for any reason. In addition to the food available in the forest and the four species-appropriate meals they were provided, all subjects received mango juice (study 1) and carrots (study 2) in their night rooms, used as our testing rooms. Water was available ad libitum in the night rooms.

**Study 1: Methods**

**Study 1: subjects**

Fifteen additional chimpanzees (7 male) were familiarized but were dropped before the experiment because they failed to reach a pre-test comprehension criterion. Eleven additional chimpanzees participated in a drinking rate preference test. Of these, 4 were dropped due to a lack of participation and 1 was dropped due to difficulty using the drinking nipple.

**Study 1: materials**

The resource used in the Eternal Fountain of Juice study was diluted mango juice (1 part 100% pure mango juice + 2 parts water; approximately 10% sugar). The drinking nipples were separated by a distance of 148cm (each 73cm from the bars separating the enclosures). Inside the apparatus cylinder was a red cork (3cm diameter, 7cm height) with an internal magnet. The cork floated up and down the vertical length of the cylinder with the level of juice, rising up as more juice dripped into the cylinder from the source juice bottle, and sinking when subjects drank juice. 20cm from the bottom of the cylinder a red threshold marker extended around the cylinder, from which a sliding external magnet held a weight tied to the plug at the bottom of the cylinder. Extending 6cm above the threshold line was a transparent red “danger zone” to increase the visual salience of the threshold. When the cork dropped fully into the danger zone and approached the threshold line the internal and external magnets connected, releasing the weight which pulled out the plug and allowed all cylinder juice as well as all future dripped juice to flow into a bucket on the ground, inaccessible to the subjects. This event represented resource collapse.

The electronic flow meters (EFM) inserted into each subject’s drinking hose had the following specifications:

B.I.O.-TECH FCH-midi-POM Art. Nr.: 97478692 with ARS-Durchflussanzeige

Impulse counter, Model 261: Art. Nr. 82202164 +82212264) including a 3mm nozzle

insert to increase the impulse detection at low rates of flow and a lithium battery

(SL-761/S).

**Study 1: electronic flow meter (EFM) calibration**

To correct for the underestimation of juice flow at low flow rates, a sample data set was collected with the Electronic Flow Meters outside of the experimental context. To do this, the experimenter manually poured 200ml of juice through the hoses with the EFMs inserted. The experimenter held the top and bottom ends of the hoses at various different heights, allowing gravity to pull the juice through the hoses at varying speeds. The time it took for the 200ml to flow through the hoses was measured, along with the final EFM readout for each test flow trial. This was repeated 34 times using a total of 12 different methods (68 trials total – 34 per EFM, (n=2)). Methods here refers to different height differentials between the two hose ends to create different rates of flow. In only one of these 12 flow methods was a chimpanzee involved, a naïve, non-subject in the experiment. This chimpanzee was involved in this method because he was able to suck juice at faster rates than gravity alone could produce. All other methods involved the experimenter pouring juice through one end of the hose and measuring the EFM timing and readout amounts without any chimpanzee involvement, by manipulating the height of the lower end relative to the higher end of the tube. The goal of this testing was to sample as wide a variety of speeds of flow as possible to measure the ranges of juice amount estimates the EFMs produced at different speeds. The faster rates of flow were the most accurate to 200ml measurements given by the EFMs. The error found was consistently in the low rates of flow (increasing in inaccuracy the lower the rate) in which the EFMs systematically underestimated the juice. Using the dataset of 34 trials we first plotted the data to get a sense of the relationship. We determined the best line of fit would be an exponential model: we modelled the amount of juice that flowed through the EFM per rotation as amount=c_0_+c_1_+c_2_^indicated flowspeed^, with c_0_, c_1_, and c_2_ being the estimated coefficients. Using R (version 3.3.2: 1), we successfully fitted an exponential model to the sample flow data set (Figure S.1) and used the coefficients of this model to correct all EFM data from the experimental trials.

We cross-validated the model to test its performance. To do so, we excluded each measure from the data, one at a time, fitted the model to the remainder of the sample, and then made a prediction for the actual amount of juice that flowed through the EFM. Finally, we compared the predicted with the actual amount, which revealed good concordance (Figure S.2).


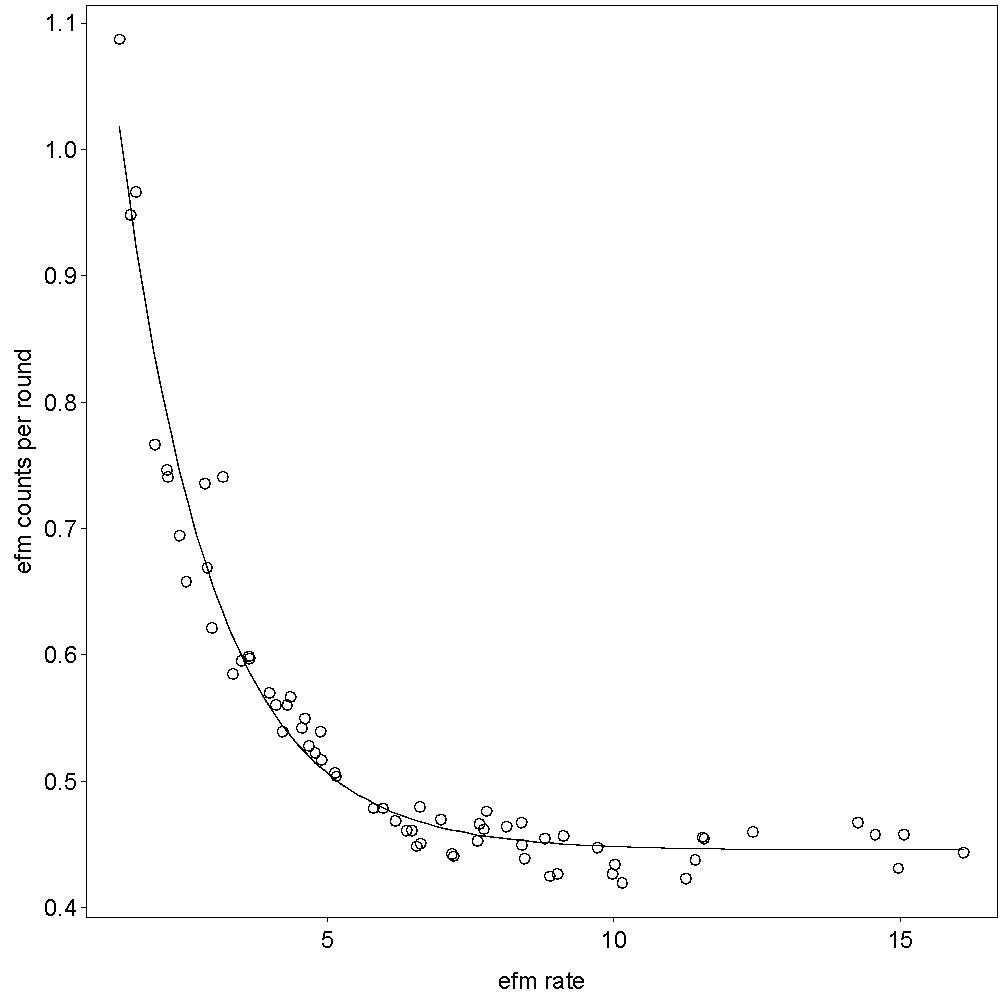


**Figure S.1 Exponential EFM correction model, with the original data points corresponding to the measured rate of flow through the EFM on the x-axis and the amount of juice per rotation of the EFM on the y-axis. This plot visualizes the inaccuracy of the EFMs, which under-estimated amounts at low rates of flow.**


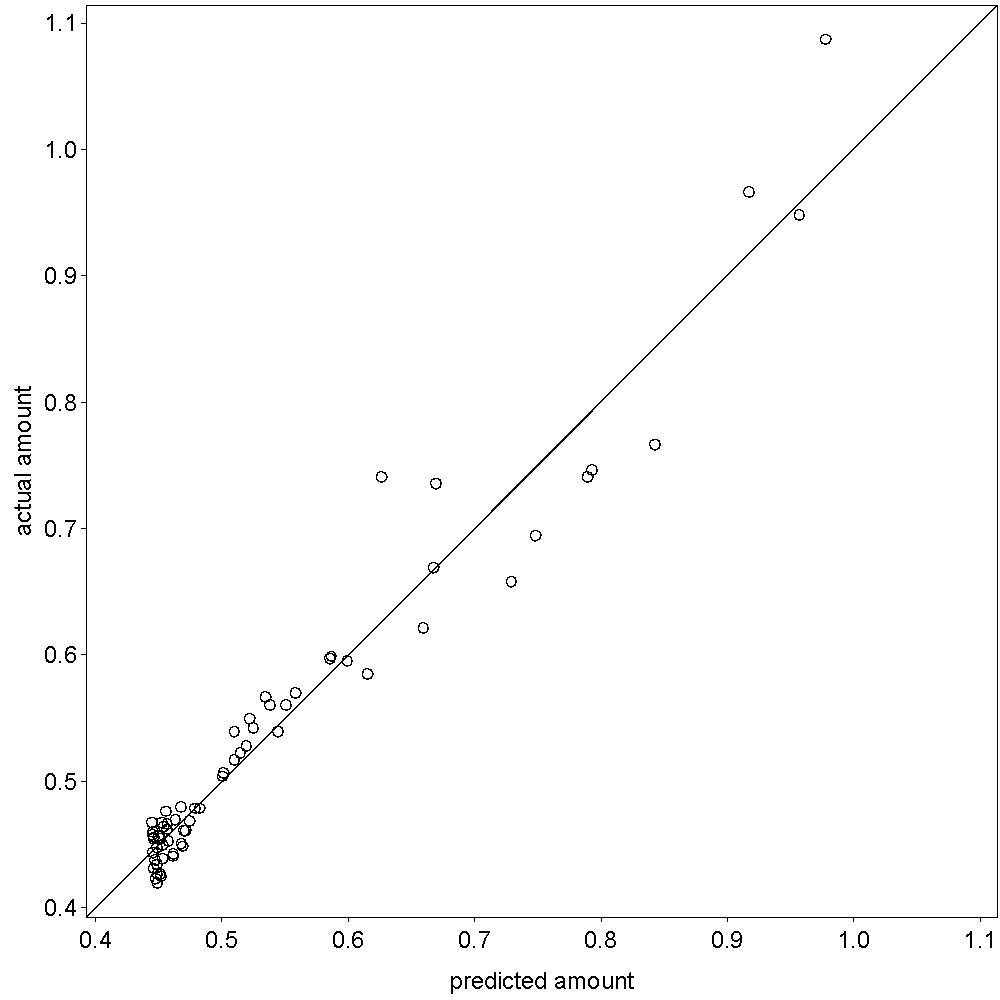


**Figure S.2. Exponential EFM correction model cross-validation: predicted amount of juice per rotation of the EFM on the x-axis against actual juice amounts on the y-axis.**

**Study 1: pre-test & familiarization**

Six subjects, naive to the apparatus and its affordances, were presented with a pre-test to determine, outside the context of a CPR dilemma, baseline drinking rate preferences with the apparatus including a full trial’s worth of juice (1800ml: 200ml starting cylinder juice + 1600ml dripping source juice). In these trials, no collapse was possible and source juice was poured by the experimenter ad libitum into the cylinder so subjects could drink at their preferred rate. Each subject received three trials, one per day for three subsequent days. Drinking duration was measured from the time they gained access to the drinking nipple to the time the cork reached the threshold after all the source juice had been poured into the cylinder.

Eighteen chimpanzees were familiarized with the full apparatus individually. Over multiple trials, the experimenter used a combination of manual inhibition (removing the drinking nipple to stop drinking and prevent collapse) as well as visual (fear grimace) and auditory (whimpers) cues to associate the cork’s presence in the danger zone with the potential for collapse. All subjects were given a series of up to ten initial sessions, three trials each, in which they waited through the dripping process of the juice renewal up to 200ml above threshold, then were given access to the drinking nipple and allowed to drink to collapse. During the second of these three trials, all subjects also experienced two manual inhibition events in which the drinking nipple was removed before collapse occurred and subjects’ attention was directed at the cylinder while the dripping process slowly raised the cork back to 200ml above threshold. Throughout these initial ten sessions the experimenter sat immediately adjacent to the cylinder and expressed visual (fear grimace) and auditory (whimpers) cues any time the cork fell into the danger zone to indicate the collapse risk. If, after all ten initial sessions, subjects continued to drink irrespective of the experimenter’s cues and the collapse potential they moved on to a set of five reduced sessions in which only one trial was given with no forced inhibition. The predicted value of the reduced sessions was to give subjects more experience with the collapse event with a costly amount of time (one day) until the next attempt. Eleven subjects still showed no signs of self-imposed inhibition after five reduced sessions and were dropped from the study. Seven subjects repeatedly stopped drinking in response to the cues provided when the cork fell into the danger zone. They were each given further familiarization sessions in which the experimenter subsequently decreased the visual, auditory, and proximity cues until each subject could sustain the juice system in the complete absence of the experimenter. Four subjects did not reliably maintain sustainable drinking patterns with reduced experimenter cues and were dropped from the study. Three subjects succeeded in passing three successive criterion test trials in which they individually accessed more than 1L per trial from the source juice before collapse. Including all familiarization sessions to the first passed criterion test, the three subjects began successfully sustaining the juice resource at 12, 19, and 30 sessions, respectively.

**Study 1: design & procedure**

Manual drip rate increases were only implemented when the drip rate fell below a visually estimated rate. Increasing the drip rate could be done once in the first 10 minutes of any trial and again during the second 10 minutes of any trial. Other than drip rate increases the experimenter was never in physical proximity to the juice system(s) or visually accessible to subjects at any time during trials, in keeping with previous methods in related tasks to avoid experimenter effects on chimpanzee behaviour (2).

The three subjects were re-paired in all three possible combinations and were tested as dyads in succession of one another, switching rooms for each cycle. To show individual success was reliable across time and testing rooms, all subjects passed a set of three criterion tests prior to each new experimental cycle.

**Study 1: coding & analysis**

Estimated drip rate was measured for the first three minutes of all trials because this was considered the most vulnerable time for competition between subjects and because we predicted that the initial drip rate established at trial start would have the biggest impact, if any, on this competition.

Gestures were defined as any hand or arm gestures produced within a meter of the bars by either subject in the direction of the partner. Gestures should culminate in a hand or arm outstretched towards the partner, which should exclude all arm out-stretches that culminate in grabbing the bars in front of or to the side of subjects, or that culminate in touching the ground. Gestures should be non-functional in the attainment of any physical goal such as reaching for something or grabbing something. Gestures should be only coded as such when a clearly visible outstretching of the arm occurs with no other discernible function other than physical movement of the hand or arm in the direction of the partner. Gestures may be accompanied by a vocalization (in which case, make note of this). Gestures may also be accompanied by a gaze in the direction of the partner (in which case make note of this).

In addition to gestures, we coded for two further categories of social events, defined as, 1) bars touches/entries: when a subject touched or inserted any part of the body between the bars separating the two subjects in the 1.5-meter experimental area, and 2) physical contact between subjects: when the two subjects made physical contact through the bars during a trial.

Inter-rater reliability was compared for 20% of trials: drinking rate preference pre-test duration *r*=1.0; collapse latency *r*=1.0; latency to full (1600ml) drip stop *r*=1.0; estimated drip rate in the first three minutes *r*=0.81; timing of manual drip increases relative to trial start *r*=1.0; partner absence bout onset, offset, and durations all *r*=1.0; EFM drinking bout onset *r*=0.99; drinking bout offset *r*=0.99 EFM calibrated drinking amounts per trial *r*=1.0; and frequency of social events: gestures *r*=1.0, bar touches *r*=0.96, and partner touches *r*=1.0.

The analysis was conducted in R (version 3.3.2: 1) using the R-package lme4 (3). For all models, we ran a series of diagnostic tests (see supplementary material for details). With the exception of model stability, these revealed no violations of the tests’ assumptions, unless otherwise stated. Due to the fact that only three individuals participated in the study, some models showed less than ideal model stability. Subsequent interpretations of the data are cautious for this reason.

Variance Inflation Factors (4) were derived using the function vif of the R-package car (5), lacking the random effects and, where applicable, the interactions. All models included maximal random slopes structures (without correlations among random intercepts and slopes) to minimize the type I error rate (6, 7) unless otherwise stated.

We compared all full models with a corresponding null model containing only control predictors, random intercepts, and random slopes (8) to test the collective effect of the test predictors on the response. Effects of individual predictors reported in the following section are based on likelihood ratio tests, after the full-null model comparisons indicated either a trend (0.1 > *P* > 0.05) or a significant effect (*P* < 0.05) of the test predictors on the response.

Because cumulative self-distraction levels appeared to increase with session duration, and cumulative synchronicity appeared to decrease with session duration, we could not test formally for the effect of these proportional behaviours on collapse latency.

**Study 1: Results**

A total of 24 social events were observed, 12 per condition, occurring in 14 out of 36 trials. A breakdown of the occurrences of each event type appears in Table S.1.

**Table S.1.** Frequency of social events according to condition.

|  | Parallel | Collective |
| --- | --- | --- |
| total frequency | 12 | 12 |
| bars touch/entry | 9 | 1 |
| partner touch | 2 | 1 |
| gesture | 1 | 10 |

The average full (1600ml) source juice drip time was 18:11 with a range of 14:34 to 22:55 (range represents trials in which the source juice dripped empty for the most conservative estimate of drip range because drip rate naturally decreased with time within trials).

We tested for the effect of condition on average individual drinking rate at the trial level (measured as the total amount of juice (ml) acquired by each individual per trial over trial duration). The sample size for this test was 68: 36 trials at the level of each individual within the three dyads; 4 individual trials were lost due to EFM technical failure. Controlling for changes in drinking rate based on experience (trial number) within each condition, the main effect of condition did not have a significant effect on drinking rate (see Fig. S.3: χ2=0.09, df=1, p=0.77). The three subjects therefore did not change their average drinking rates between the collective and the parallel conditions suggesting they were not implementing an equal distribution strategy.


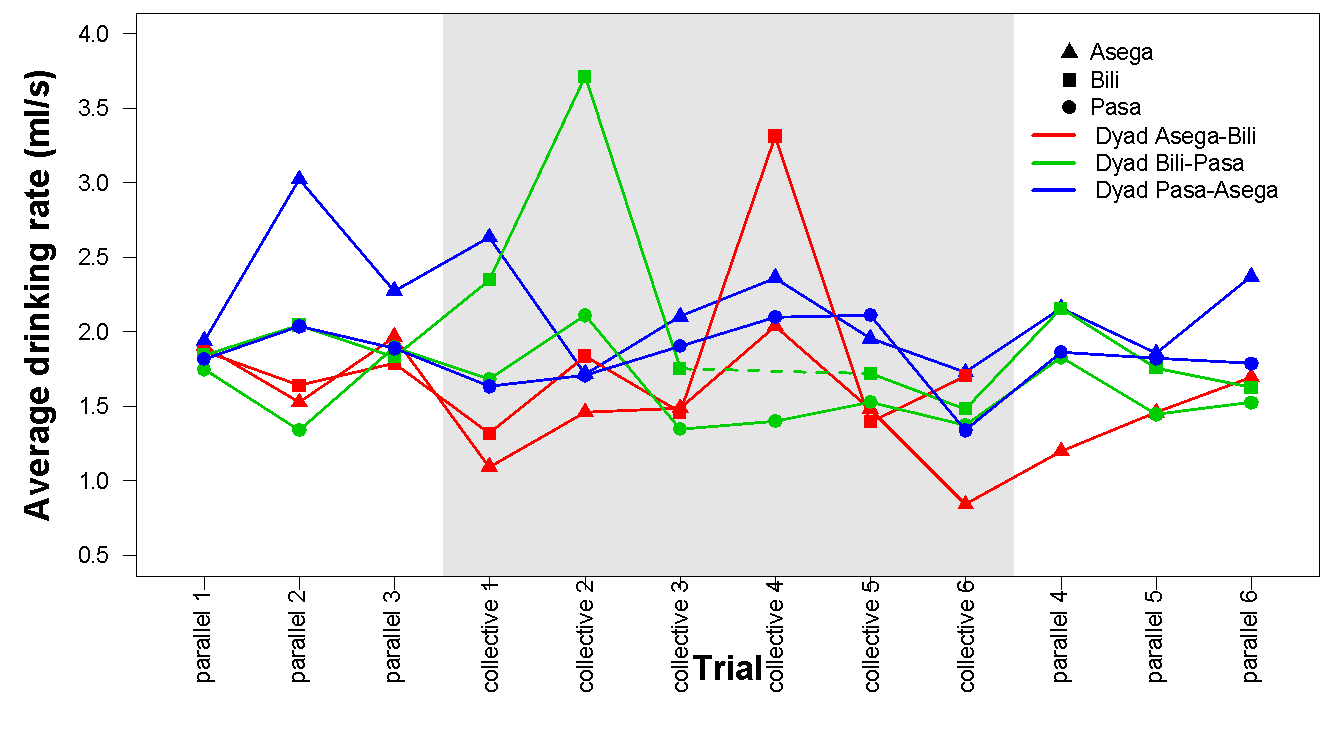
**Figure S.3. Average drinking rates per individual (symbols) within each dyadic pairing (colours) per trial. From left to right, all experimental trials (A-B-A) in chronological order, with collective condition trials highlighted in the central grey area. EFM failure caused the loss of 4 trials; these are represented by the dotted green lines for Bili in dyad BiliPasa and the lack of a red line for Bili in dyad AsegaBili for the last three parallel trials.**

We also tested for the effects of partner presence and absence from the drinking nipple area (>1m) on drinking rate within trials. Here, we measured the amount of juice acquired by subjects during bouts of partner presence and partner absence. We predicted an interaction between condition and bout type (i.e. absence or presence of partner) on drinking rate within bouts, given that the absence or presence of a partner in the collective condition indicates whether or not the partner is able to access the shared juice source at that time. We found no evidence of an interaction between condition and bout type (χ2 = 0.82, df = 1, p = 0.36) however, across both conditions, drinking rate tended to be higher during bouts of partner presence than during bouts of partner absence (non-significant trend: χ2 = 3.73, df = 1, p = 0.054; see Figure S.4). The timing of drinking bout onset within trials relative to the time the drip rate was last increased (i.e. trial start or any subsequent manual drip rate increases by the experimenter) had a highly significant effect (χ2 = 7.578, df = 1, p < 0.01), indicating that subjects were flexibly adjusting their within-trial drinking patterns according to drip rate so as to strategically avoid collapse.

Subjects slowed their drinking rate, in both conditions, in response to their partner’s absence. This finding is in line with our prediction that drinking in close proximity to a partner would cause a socially facilitated increase in resource acquisition rate (9, 10). This is striking, given that subjects could also have increased their drinking rate in the collective condition when their partners were absent in order to maximize their juice intake while their partner was not around to increase the collapse risk (however, this may have been observable with a larger sample size).


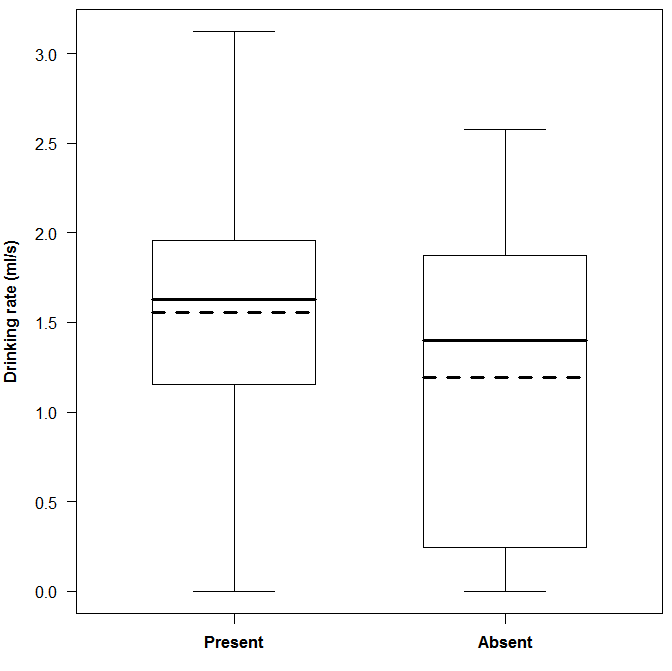


**Figure S.4. Drinking rate according to the presence or absence of each subject’s partner. Solid lines represent median drinking rates per bout type; dotted lines represent predicted drinking rates according to the model. Boxes depict 25-75th percentiles of drinking rates per bout type; whiskers represent 2.5th and 97.5th percentiles.**

For individual juice amounts consumed per session, see Table S.2, below, and for the inequality analysis, see Figure S.5., below.

**Table S.2.** EFM estimated individual juice amounts consumed per session. Empty cells (4) due to EFM failure.

| Session: | Dyad Asega-Bili | | Dyad Bili-Pasa | | Dyad Pasa-Asega | |
| --- | --- | --- | --- | --- | --- | --- |
|  | Asega | Bili | Bili | Pasa | Pasa | Asega |
| Parallel 1 | 778.0ml | 901.6ml | 1767.9ml | 1384.7ml | 1605.4ml | 1456.5ml |
| Parellel 2 | 1278.6ml | 1999.3ml | 448.2ml | 241.3ml | 277.6ml | 424.4ml |
| Parellel 3 | 1359.0ml | 1720.1ml | 1708.7ml | 1622.5ml | 1151.7ml | 1251.5ml |
| Collective 1 | 720.4ml | 1243.2ml | 399.6ml | 246.9ml | 255.6ml | 368.1ml |
| Collective 2 | 343.2ml | 589.8ml | 328.6ml | 117.4ml | 615.3ml | 473.8ml |
| Collective 3 | 258.1ml | 448.8ml | 438.7ml | 275.5ml | 568.3ml | 412.3ml |
| Collective 4 | 144.1ml | 376.0ml | - | 276.8ml | 372.8ml | 295.6ml |
| Collective 5 | 438.3ml | 719.3ml | 351.4ml | 210.7ml | 367.1ml | 256.0ml |
| Collective 6 | 116.5ml | 1838.0ml | 499.4ml | 365.6ml | 353.1ml | 1242.0ml |
| Parallel 4 | 824.0ml | - | 626.7ml | 152.2ml | 911.1ml | 872.1ml |
| Parallel 5 | 1129.5ml | - | 1778.3ml | 1124.8ml | 1773.6ml | 1116.6ml |
| Parallel 6 | 1503.8ml | - | 1793.3ml | 1358.6ml | 705.0ml | 761.4ml |


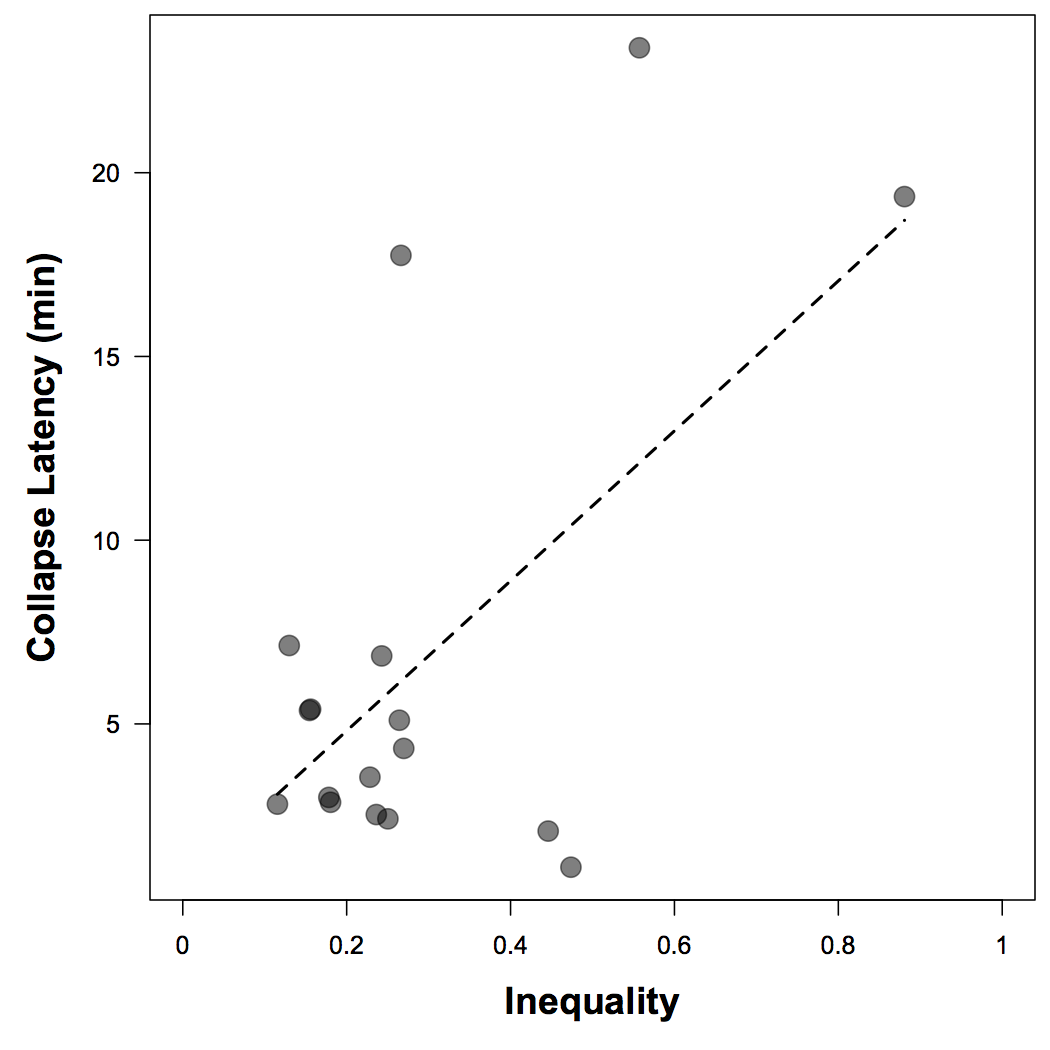


**Figure S.5. Inequality of juice distribution between partners per collective condition trial, as a function of collapse latency (minutes). Dotted line represents trend predicted from model. Inequality measure is derived by dividing the absolute difference in amounts of juice drunk by each partner per trial by the total juice drunk by the dyad.**

All collective condition gestures were observed in one dyad, the majority of which occurred in this dyad’s final collective condition trial, which was also their most successful (although this dyad performed overall more poorly than the other two dyads). See attached video.

**Table S.3. Study 1 Model descriptions**

| **Question** | **Dependent measure** | **Test predictors** | **Control predictors** | **Random intercepts** | **Random slopes** | **R-function** | **Error structure** | **Model diagnostics** | |
| --- | --- | --- | --- | --- | --- | --- | --- | --- | --- |
| Did condition, trial number, or drip rate affect success? | Collapse latency^L^  N=35 trials | Condition*Trial^z^,  Drip rate^z^ |  | Dyad | Condition, drip rate^z^, & trial no.^z^. within dyad | lmer | Gaussian | | Variance inflation factors (VIFs), stability & residuals |
| Did drinking rate decrease from the parallel to the collective condition? | Average individual dinking rate^L^ per trial  N=68 | Condition | Trial no.^z^ | Trial ID, Individual, Dyad | Condition & Trial no.^z^ within dyad & individual | lmer | Gaussian | | VIFs, stability & residuals |
| Did partner presence affect drinking rate? | Individual drinking rate per partner’s presence/ absence bout  N=299 | Condition*Bout type (i.e. absent or present) | Time since drip rate increase, Trial no.^z^ | Bout, Individual,  Dyad, trial, ID of trial segment since drip rate increase (block) | Condition*Bout type, trial no.^z^ , & time since drip rate increase within dyad and individual; Bout type & time since drip rate increase within trial; Time since drip rate increase within block | lmer | Gaussian | | VIFs, stability & residuals |
| Did inequality affect success in the collective condition? | Collapse latency  N=17 | Inequality of juice amounts^z^ |  | Dyad | Inequality of juice amounts^z^ within dyad | lmer | Gaussian | | Stability & residuals |
| Did direction and degree of inequality predict identity of collapser | y/n drinking at collapse per individual  N=34 | Individual inequality proportion (Inequality^z^) |  | Dyad, Individual, Trial | Inequality^z^ within dyad, individual, and trial | glmer | Binomial | | Stability |

^L^ Denotes log-transformation to normalize residuals

* Denotes an interaction, implies inclusion of both main effects

^z^ Denotes a z-transformed numeric variable

**Table S.4. Study 1 Full-null model comparisons**

| Question | *X^2^* | DF | *p* |
| --- | --- | --- | --- |
| Did condition, trial number or drip rate affect success? | 8.253 | 4 | 0.083 |
| Did drinking rate decrease in the collective condition? | 0.088 | 1 | 0.767 |
| Did partner presence affect drinking rate? | 5.669 | 2 | 0.059 |
| Did juice inequality affect success in the collective condition? | 3.340 | 1 | 0.068 |
| Did direction and degree of individual inequality predict identity of collapse? | 0.060 | 1 | 0.806 |

**Table S.5. Study 1 Final model summaries**

| Question | Predictors | Estimate | Standard Error | *X^2^* | DF | *p* |
| --- | --- | --- | --- | --- | --- | --- |
| Did condition, trial number or drip rate affect success? | Intercept | 2.463 | 0.203 |  |  |  |
|  | Condition | -1.006 | 0.216 | 6.934 | 1 | 0.008 |
|  | Trial no.^z^ | 0.120 | 0.109 | 1.202 | 1 | 0.273 |
|  | Drip rate^z^ | -0.014 | 0.111 | 0.014 | 1 | 0.905 |
| Did drinking rate decrease in the collective condition? | Intercept | 0.585 | 0.101 |  |  |  |
|  | Condition | -0.033 | 0.074 |  |  |  |
|  | Trial no.^z^ | -0.052 | 0.029 |  |  |  |
| Did partner presence affect drinking rate? | Intercept | 1.254 | 0.167 |  |  |  |
|  | Condition^Prll^ | -0.115 | 0.105 | 1.041 | 1 | 0.308 |
|  | Bout type^Abs^ | 0.242 | 0.100 | 3.727 | 1 | 0.054 |
|  | Time since drip inc. | -0.408 | 0.055 | 7.578 | 1 | 0.006 |
|  | Trial no.^z^ | -0.068 | 0.053 | 1.616 | 1 | 0.204 |
| Did inequality affect success in the collective condition? | Intercept | 6.768 | 1.264 |  |  |  |
|  | Inequality^z^ | 3.975 | 1.303 | 3.340 | 1 | 0.068 |
| Did direction and degree of individual inequality predict identity of collapse? | Intercept | 1.458 | 1.103 |  |  |  |
|  | Individual Inequality^z^ | -0.303 | 1.293 | 0.060 | 1 | 0.806 |

^z^ denotes z-transformation

* denotes interaction

^Prll^ denotes the parallel condition as reference category

^Abs^ denotes absent as reference category

**Study 1 & 2: comparison between study designs**

There were several differences between the CPR paradigm used in study 1 and the paradigm used in study 2. These differences were implemented in study 2 with the goal of making the task simpler to comprehend. First, the condition comparison difference between studies 1 & 2 was such that each parallel system in study 1 contained as much of the resource as the one shared system in the collective condition, whereas in study 2, the two parallel systems were combined to make the collective system. Second, the renewing resource system in study 1 had a visually transparent accumulation mechanism intended to decrease the complexity of the behavioural contingency between one’s own feeding behaviour and the future potential for resource accumulation. Additionally, the resource collapse mechanism in study 2 involved an active decision to reach out and grab a piece of the apparatus, whereas collapsing the juice fountain in study 1 was a much more passive act – one had to simply *continue* to drink from the renewing stock of juice. Last, we lowered the value of the food resource from mango juice in study 1 to carrot pieces in study 2, which we predicted would aid in making the task easier.

The comparison between the parallel and collective conditions also differed between study 1 and 2. In study 1, the collective condition essentially renewed at half the per-individual rate as the juice systems of the parallel condition. In contrast, the carrot system of study 2 renewed at the same per chimpanzee rate in both conditions.

Take, as an example, a population of fish in a lake. Anyone fishing on this lake for personal profit will be incentivized to maximize collection, however if all fishers indeed maximize their catch, over time the population may decline to the extent that fish reproduction slows or even collapses entirely. Therefore, to successfully sustain the fish resource over the long-term, fishers must limit their individual catch, while enforcing the same on potential free-riders. Using this analogy, study 2 was the equivalent of a shift from two independently functioning fisheries on two small lakes, moving to one collective fishery on a third lake with a reproduction rate that is twice as fast as that of the original two lakes, such that each fisher in the collective fishery can continue fishing at the same rate as they fished independently, without risking collapse. The comparison of the two conditions in study 2 differed only with respect to the interdependence of the task. Functionally, this also meant that the same quantity of carrot pieces was physically present in the testing area for all parallel and collective trials so any effects of simple arousal at the sight of food of various quantities would be mitigated in study 2. The fact that success was significantly lower in the collective condition of study 1 could, therefore, be interpreted as a result of an increasingly difficult delay of gratification task in the collective relative to the parallel condition, however the comparison between these two conditions in study 2 rules out this interpretation. In both studies, the lower rates of success in the collective condition indicate the difficulty of the social dilemma involved. Additionally, both studies provide evidence that outcome interdependence – and hence competition – hinders delay of gratification skills in chimpanzees.

**Study 2: Methods**

**Study 2: pre-tests & familiarization**

Figure 7a: For clarity of display E1 is not pictured on platform with E2 in the collective condition although she was present for trials.

In the first (Phase 1) familiarization session, each subject had the chance to collect 80 pieces of carrot falling two per 10-second interval across two trials. Importantly, no carrot pieces were baited on the wooden rod for these trials. If subjects collapsed the apparatus in the first trial, the carrot accumulation process continued but pieces accumulated outside the feeding area. These remaining pieces were then collected by the experimenter after all 80 had fallen and the remaining pieces were then dropped at the same rate in a second trial, also continuing until all pieces had been dropped irrespective of collapse. The experimenter then baited the wooden rod with 5 pieces of carrot, allowing subjects to pull the baited rod three times, causing chute collapse, without pieces accumulating from the chute. In the second session, the two accumulation trials were presented after the three rod pulls.

The first (Phase 2) familiarization session included two trials of lower difficulty: 20 dropping iterations with 4 baited rod pieces. Subsequent sessions had only one trial but were more difficult: 40 dropping iterations, 8 baited rod pieces. If two subsequent sessions resulted in collapse before all 40 dropping iterations a third motivation-helper session was offered in which no carrot pieces were baited on the rod; if sustaining behaviour improved in the helper session then normal sessions were again presented thereafter, however, if subjects still collapsed the apparatus in the absence of baited pieces in the helper session, subsequent helper sessions were offered until a full cycle of 40 dropping iterations could be sustained before returning to normal familiarization sessions with a baited rod.

17 subjects reached the first fully sustained 10 second interval trial after an average of 4.4 sessions. 16 subjects participated in the experimental cycle. Subjects took part in only one dyadic pairing for this experiment and as such only an even number of subjects could participate.

Sixteen subjects were paired into dyads based on keeper recommendations of tolerance to avoid unnecessary stress or aggression.

All eight dyads were then tested for tolerance and dominance in a set of 4 tolerance pre-tests. Tolerance, defined as the propensity that individuals’ will share a common food source (11), has been shown to moderate dominance differences between partners in collaborative tests (11, 12). Each tolerance test followed the same procedure: dyadic subjects were simultaneously allowed entry into their adjacent rooms. In front of both subjects’ feeding holes, in the absence of the apparatus, was a matrix of 80 carrot pieces, equally accessible to both subjects and therefore potentially monopolizable.

**Study 2: design & procedure**

E1 and E2 were behind a curtain and therefore never visible to subjects during any experimental trials to minimize experimenter effects on subjects’ performance. They operated carrot dropping tools on sticks to avoid any parts of experimenters’ bodies (e.g. hands) from being visible during trials. This was also the case for all familiarization trials so the subjects were never exposed to any visual causal association between the dropping carrots and the experimenters.

**Study 2: coding & analysis**

Tolerance pre-tests were coded for two behavioural measures. The first was inequality of access to the shared 80-piece carrot matrix between partners. The total number of pieces accessed by each subject was counted and measured against an equitable 50-50% split to determine a proportion of inequality per trial. The individual with a higher aggregate amount of carrot pieces accessed over the four tolerance trials was determined to be the dominant individual. The second measure was the latency of the subordinate individual to enter the feeding hole nearest to his/her partner’s enclosure. We standardized (mean=0, S.D.=1) both measures and combined their averages for a composite co-feeding tolerance level for each dyad. In this way, identifying the dominant and subordinate individual in each dyad allowed us to take into account between-individual social relationships and the tolerance levels allowed us to explore social difference between dyads.

Twenty percent of all tolerance pre-tests and experimental trials were re-coded by a second coder, blind to the predictions of the study. Coding comparisons revealed high inter-rater reliability: collapser ID had 100% agreement; collapse latency *r*=1.0; latency of subordinate to enter partner-near feeding hole *r*=1.0; and proportion inequality *r*=0.94.

As with study 1, the analysis for study 2 was conducted in R (version 3.3.2: 1) using the R-package lme4 (3).We fitted two models, the assumptions of which were verified using a set of diagnostic tests including Variance Inflation Factors (VIFs: function vif of the R-package car (5)), over-dispersion, and model stability. No issues were discovered to lead us to conclude the assumptions of either model had been violated (see Appendix II for details). Both models included maximal random slopes structure to minimize the type I error rate (6, 7), unless otherwise stated. As with study 1,we compared both full models with a corresponding null model containing only control predictors, random intercepts, and random slopes (8) to test the collective effect of the test predictors on the response. Effects of individual predictors reported in the following section are based on likelihood ratio tests, after the full-null model comparisons indicated either a trend or a significant effect of the test predictors on the response.

To investigate differences in collapse latency between the independent parallel condition and the interdependent collective condition, we fitted a GLMM (13). The test predictors (14) with fixed effects included condition (levels: parallel & collective), as well as the (z-transformed; 15) quantitative predictors of tolerance level, and trial number (1-8) to test for a learning effect across trials. We also included random slopes for trial number within session since one session represented data from two trials, and likewise for condition and trial number within dyad, and the interaction between trial number and condition within the dyad. Because we predicted that dyadic tolerance level, condition, and trial number may differentially affect the collapse latency, we included a three-way interaction of the three test predictors as well as all three possible two-way interactions between them. The model controlled for the fixed effect of order of conditions.

To test whether the identity of the collapser (i.e. subordinate or dominant) was influenced by dyadic co-feeding tolerance we also fitted a Generalized Linear Mixed Model (GLMM; 13) with binomial response distribution; the response variable here was whether or not the subordinate caused collapse per trial. Test predictors included fixed effects for condition (levels: parallel & collective) and the (z-transformed) quantitative predictor of tolerance level. Because we predicted that the tolerance of the dyad could differentially affect the likelihood of the subordinate causing the collapse depending on the condition, we included a 2-way interaction between tolerance and condition. Here we also controlled for the fixed effect of order of conditions. As with the collapse latency model, we included a random intercept for session because each testing session contained two trials of the same condition for each dyad. We also included a random intercept for dyad and random slopes for condition within dyad.

**Study 2: Results**

**Table S.6. Study 2 Model descriptions**

| **Question** | **Dependent measure** | **Test predictors** | **Control predictors** | **Random intercepts** | **Random slopes** | **R-function** | **Error structure** | **Model diagnostics** |
| --- | --- | --- | --- | --- | --- | --- | --- | --- |
| How did condition, trial number, and tolerance affect success? | Collapase latency^L^  N=128 | Condition*Trial.no^z^*Tolerance^z^ | Order of conditions | Session, Dyad | Trial.no^z^ within session; condition* Trial.no^z^ within dyad | lmer | Gaussian | Residuals, stability, VIFs |
| How likely was the subordinate to cause collapse in each condition? | y/n subordinate cause collapse per trial  N=128 | Condition*Tolerance^z^ | Order of conditions | Session, Dyad | Condition within dyad | glmer | Binomial | VIFs, stability |

^L^ Denotes log-transformation to normalize residuals

* Denotes an interaction, implies inclusion of both main effects

^z^ Denotes a z-transformed numeric variable

**Table S.7. Study 2 Full-null model comparisons**

| Question | *X^2^* | DF | *p* |
| --- | --- | --- | --- |
| How did condition, trial number, and tolerance affect success? | 22.699 | 1 | <0.001 |
| How likely was the subordinate to cause collapse in each condition? | 7.537 | 3 | 0.057 |

**Table S.8. Study 2 Final model summaries**

| Question | Predictors | Estimate | Standard Error | *X^2^* | DF | *p* |
| --- | --- | --- | --- | --- | --- | --- |
| How did condition, trial number, and tolerance affect success? | Intercept | 5.720 | 0.380 |  |  |  |
|  | Condition^Prll^ | -1.301 | 0.325 |  |  |  |
|  | Trial.no^z^ | 0.198 | 0.114 |  |  |  |
|  | Tolerance^z^ | 0.395 | 0.271 |  |  |  |
|  | Order of Conditions | -1.036 | 0.534 | 3.083 | 1 | 0.079 |
|  | Condition* Trial.no^z^ | -0.469 | 0.184 | 5.471 | 1 | 0.019 |
|  | Condition*  Tolerance^z^ | -0.976 | 0.326 | 5.996 | 1 | 0.014 |
| How likely was the subordinate to cause collapse in each condition? | Intercept | 0.096 | 0.382 |  |  |  |
|  | Condition^Prll^ | -1.023 | 0.640 |  |  |  |
|  | Tolerance^z^ | 0.376 | 0.285 |  |  |  |
|  | Order of Conditions | 0.343 | 0.524 | 0.415 | 1 | 0.519 |
|  | Condition*  Tolerance^z^ | 1.176 | 0.717 | 3.101 | 1 | 0.078 |

^z^ denotes z-transformation

* denotes interaction

^Prll^ denotes the parallel condition as reference category

References

1. R Core Team. R: A language and environment for statistical computing. R Foundation for Statistical Computing, Vienna, Austria. URL <https://www.r-project.org/>. 2016.

2. Beran MJ, Evans TA. Maintenance of delay of gratification by four chimpanzees (Pan troglodytes): the effects of delayed reward visibility, experimenter presence, and extended delay intervals. Behav Processes. 2006;73(3):315-24.

3. Bates D, Mächler M, Bolker B, Walker S. Fitting Linear Mixed-Effects Models Using lme4. Journal of Statistical Software. 2015;67(1).

4. Field A. Discovering statistics using SPSS. 2009 London. SAGE. ISBN: 9781847879073; 2005.

5. Fox J, Weisberg S. Multivariate linear models in R. Sage Publications, Thousand Oaks, CA; 2011.

6. Schielzeth H, Forstmeier W. Conclusions beyond support: overconfident estimates in mixed models. Behavioral Ecology. 2009;20(2):416-20.

7. Barr DJ, Levy R, Scheepers C, Tily HJ. Random effects structure for confirmatory hypothesis testing: Keep it maximal. Journal of memory and language. 2013;68(3):255-78.

8. Forstmeier W, Schielzeth H. Cryptic multiple hypotheses testing in linear models: overestimated effect sizes and the winner's curse. Behavioral Ecology and Sociobiology. 2011;65(1):47-55.

9. Engelmann JM, Herrmann E, Tomasello M. The effects of being watched on resource acquisition in chimpanzees and human children. Animal cognition. 2016;19(1):147-51.

10. Evans TA, Perdue BM, Parrish AE, Menzel EC, Brosnan SF, Beran MJ. How is chimpanzee self-control influenced by social setting? Scientifica. 2012;2012.

11. Melis AP, Hare B, Tomasello M. Engineering cooperation in chimpanzees: tolerance constraints on cooperation. Animal Behaviour. 2006;72(2):275-86.

12. Schneider A-C, Melis AP, Tomasello M. How chimpanzees solve collective action problems. Proceedings of the Royal Society of London B: Biological Sciences. 2012:rspb20121948.

13. Baayen RH. Analyzing linguistic data: A practical introduction to statistics using R: Cambridge University Press; 2008.

14. Mundry R. Statistical issues and assumptions of phylogenetic generalized least squares. Modern phylogenetic comparative methods and their application in evolutionary biology: Springer; 2014. p. 131-53.

15. Schielzeth H. Simple means to improve the interpretability of regression coefficients. Methods in Ecology and Evolution. 2010;1(2):103-13.
